# Root Meristem Maintenance Mechanisms are Key to Plant Defense Against Nanoplastics

**DOI:** 10.1002/advs.202511837

**Published:** 2025-08-26

**Authors:** Sirui Ma, Zhengdong Hua, Chenglong Tang, Xinran Qiu, Ling Ding, Xujun Liang, Yuzhou Zhang, Xuetao Guo

**Affiliations:** ^1^ College of Natural Resources and Environment Northwest A&F University Yangling Shaanxi 712100 China; ^2^ State Key Laboratory of Crop Stress Resistance and High‐Efficiency Production College of Life Sciences Northwest A&F University Yangling Shaanxi 712100 China

**Keywords:** auxin, gravitropism, nanoplastics, phytotoxicity, polar transport

## Abstract

The pervasive prevalence of nanoplastics in environment poses a challenge that threatens ecosystem and agricultural production. Despite their ubiquity, the determinants of nanoplastics phytotoxicity and the mechanisms through which plants defend against this phytotoxicity remain poorly understand. In this study, it is demonstrated that the phytotoxicity of nanoplastics is inversely correlated with particle size. Specifically, polystyrene‐nanoplastics sized at 20 nm dramatically inhibit root growth in *Arabidopsis*, while larger particles (100 to 1000 nm) have minimal effects. Mechanistically, these small nanoplastics primarily target the root meristem (RM), disrupting cell integrity and inhibiting cell division, which impairs root development. Plants employ two key defense strategies to counteract this toxicity: i) upregulating genes associated with RM maintenance and ii) accumulating auxin in the roots by inhibiting the auxin efflux transporter PIN2‐dependent efflux of auxin, thereby reducing upward transport. However, this defensive response comes at a cost, as it also impairs root gravitropism, a critical process for plant adaptation to environmental changes. These findings provide valuable insights into the mechanisms of nanoplastic‐induced phytotoxicity and plant defense, establishing a foundation for the development of biosafe plastic products and strategies to genetically enhance plant resistance to tiny nanoparticle exposure by optimization of intrinsic detoxification pathways.

## Graphical abstract

Small nanoplastics can disrupt cell integrity and inhibit cell division in meristem zone of plant root. The plants can counteract the cell damage by upregulating genes associated with root meristem maintenance and increasing auxin accumulation by PIN2 transport inhibition. The defense strategies enhance stress tolerance but impair the gravitropic growth of plants.

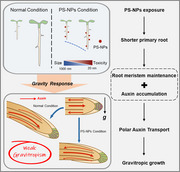

## 1 Introduction

To address pressing environmental challenges such as global warming, desertification and resource scarcity, plastic products have been widely adopted across various sectors, including agriculture, where they have significantly enhanced crop yields and quality.^[^ ^1^, ^2^, ^3^, ^4^ ^]^ However, the accumulation of unrecycled plastic residues in agricultural soil, stemming from inadequate post‐harvest recycling of plastic products, has become a serious environmental issue.^[^ ^5^, ^6^ ^]^ The breakdown and degradation of these residues, particularly under the greenhouse conditions, leads to the formation of micro‐ and nanoplastic particles.^[^ ^7^, ^8^ ^]^ Due to their smaller size and higher surface area, these particles act as active carriers for organic pollutants and pathogenic microorganisms.^[^ ^9^, ^10^ ^]^ Agricultural soils act as a primary sink for micro‐ and nanoplastic particles, accumulating at rates that surpass even those in oceanic reservoirs,^[^ ^11^, ^12^ ^]^ with contamination levels potentially reaching 4‐23 times higher than those found in marine ecosystem.^[^ ^13^ ^]^ The presence of these particles in soil threatens the sustainability of agricultural production by altering soil physicochemical properties and disrupting soil biota community.^[^ ^14^, ^15^, ^16^ ^]^

As primary producers in food chain, plants play a fundamental role in ecosystem material cycling. Micro‐ or nanoplastics can adversely affect plant growth and development in several ways. For example, they can decrease soil porosity, hindering moisture uptake and lowering seed germination rates.^[^ ^17^, ^18^, ^19^, ^20^ ^]^ Nanoplastics may penetrate seed coats and trigger the plant's antioxidant defense system.^[^ ^21^ ^]^ Irregular plastic fragments may entangled young plant roots, stunting vegetative growth,^[^ ^22^ ^]^ while smaller nanoplastics adhere to root surfaces due to their higher surface area and greater interaction with root exudate.^[^ ^23^ ^]^ These particles can enter the plant's xylem vessels through the apoplastic pathway in the Casparian strip and reach aboveground tissues via the vascular system.^[^ ^24^, ^25^ ^]^ Furthermore, plant leaves can capture micro‐ and nanoparticles from atmospheric deposition, which can then enter mesophyll cells through stomata or the cuticle.^[^ ^26^ ^]^ Once absorbed, nanoplastics may undermine the sustainability of agricultural practices, posing a significant threat to plant health and productivity.

Plants exhibit multi‐level responses to micro‐ and nanoplastics, which affect individual tissues, organs, and various physiological and biochemical processes.^[^ ^21^, ^27^ ^]^ Studies have shown that plastics can alter plant morphology, reduce biomass,^[^ ^28^, ^29^ ^]^ and induce oxidative stress by increasing reactive oxygen species (ROS), disrupting the expression of genes associated with metabolic enzymes, and affecting overall plant metabolism.^[^ ^30^, ^31^, ^32^ ^]^ For example, Hussain et al. compared the effects of different sizes of PS‐NPs on citrus plants and found that small‐sized nanoplastics (20 nm) had a negative impact on the root structure and nutrient content of plants compared to the 50 nm treatment.^[^ ^33^ ^]^ Gao et al. investigated the effects of PS particles with different sizes (0.1, 1, and 10 µm) on wheat seedlings and found that plants only absorb small‐sized nanoparticles, and particle size was the main factor causing oxidative damage and changing endogenous homeostasis.^[^ ^34^ ^]^ Studies on the effects of nanoplastics on microalgae have shown that 2 µm microplastics affect their photosynthesis, while 0.1 µm PS‐NPs damage the integrity of the cell wall.^[^ ^35^ ^]^ In order to reduce the stress caused by nanoplastics, plants present various adaptations from surface structure to genetic level, such as developing more lateral roots and root hairs to improve nutrient absorption,^[^ ^27^ ^]^ reinforcement on secondary cell wall to reduce uptake of nanoplastics.^[^ ^36^ ^]^ It produces related antioxidant enzymes such as peroxidase (POD) to protect cells from oxidative damage.^[^ ^37^ ^]^ For instance, Yin et al. found that PS‐NPs exposure activated the biosynthesis pathway of lignin and suberin in roots, forming a low permeability barrier to effectively block the invasion of nanoplastics.^[^ ^38^ ^]^ Plant tropisms—directional growth in response to environmental stimuli, such as gravity and light,^[^ ^39^, ^40^ ^]^ are critical for plant adaption to various abiotic stresses, including drought, salinity and hypoxia.^[^ ^41^, ^42^, ^43^ ^]^ Among these, gravitropism, which directs root growth downward, shapes root system architecture (RSA) and influences agricultural traits such as water and nutrient uptake and drought tolerance.^[^ ^44^ ^]^ Despite its importance, most current research has focused on morphological, physiological, biochemical, and metabolic responses to micro‐ and nanoplastics. Little is known about how these pollutants impact plant tropisms, especially gravitropism.

*Arabidopsis thaliana* is well‐characterized model plant, providing an ideal system for studying these effects due to its simple genome, small stature, speedy reproductive cycle, and ease of observation.^[^ ^23^, ^45^ ^]^ Research on *Arabidopsis* has greatly advanced our understanding of plant growth and environmental responses, contributing to both food production and environmental protection. Polystyrene (PS), a synthetic aromatic polymer, is commonly synthesized as nanoparticles, which are widely used in scientific research due to their well‐defined size, shape, and surface properties. Polystyrene nanoplastics (PS‐NPs) discard from packing, food containers and other fields have become an important factor in the global plastic pollution crisis.^[^ ^46^ ^]^ In environmental studies, PS‐NPs are often used as model particles to investigate the behavior and effects of nano‐sized plastics on biological systems.^[^ ^47^, ^48^, ^49^ ^]^ Their interactions with living organisms, including plants and animals, are of significant interest because they mimic the effects of various environmental nanoplastics. In this study, we systematically explore the effects of different‐sized PS‐NPs on root developmental growth in *Arabidopsis*, as well as plant defense response to small PS‐NPs. We aim to identify key pathways and potential mechanisms by which PS‐NPs of varying sizes differentially regulate root growth and its gravitropic response.

## 2 Results

### 2.1 The Phytotoxicity of PS‐NPs Compromises Root Growth in a Particle‐Size‐Dependent Manner

**Figure 1:**
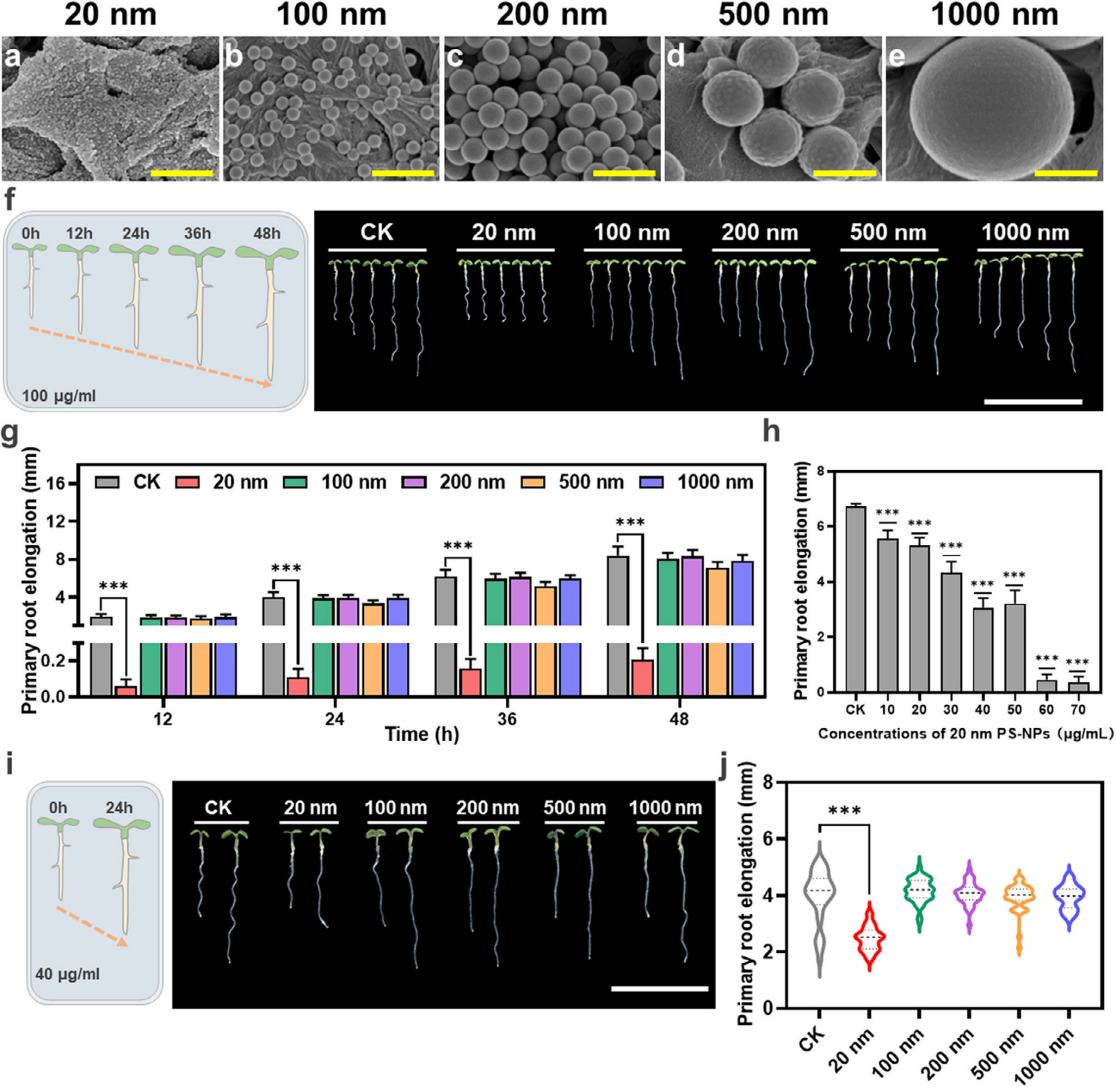
Characterization of PS‐NPs of various sizes and their impact on primary root growth in *Arabidopsis thaliana*. a–e) SEM images of different PS‐NPs with diameters of 20 nm (a), 100 nm (b), 200 nm (c), 500 nm (d), 1000 nm (e). Scale bar, 500 nm. f) Phenotypic images depicting primary root growth in *Arabidopsis* seedlings exposed to 100 µg mL^−1^ PS‐NPs of varying sizes over time. Images were captured every 12‐h over a 48‐h period. Scale bar, 1.5 cm. g) Quantitative analysis of primary root elongation at different time points under treatment with 100 µg ml^−1^ PS‐NPs. h) Changes in primary root elongation after 24 h of treatment with different concentrations of 20 nm PS‐NPs. i) Phenotypic images depicting primary root growth at an exposure of 40 µg ml^−1^ PS‐NPs for 24 h. Scale bar, 1 cm. j) Violin plot illustrating root elongation length in (i). Data are presented as means ± SD. Statistical analysis was conducted using one‐way ANOVA followed by Dunnett's multiple comparisons test. * *p* < 0.05, **P < 0.01, and *** *P* < 0.001.

To evaluate the environmental impact and toxicity of PS‐NPs of varying sizes on plant growth and development, we analyzed five different types of PS‐NPs with sizes of 20, 100, 200, 500, and 1000 nm for analysis. Electron microscope revealed uniform dimensions and a sphere‐shaped distribution of these plastic particles (**Figure** **1**a–e; Figure S1a, Supporting Information). Dynamic light scattering confirmed their effective dispersion in deionized water (Figure S1b–f and Table S2, Supporting Information). Our phenotypic analysis showed that *Arabidopsis* treated with the smallest PS‐NPs (20 nm) at a concentration of 100 µg ml^−1^ exhibited a significant inhibition in root growth compared to untreated controls, whereas those treated with PS‐NPs ranging from 100 to 1000 nm displayed no noticeable growth defects (Figure 1f,g). These findings indicate that smaller PS‐NP (20 nm) substantially inhibit plant development due to their heightened phytotoxicity.

To further confirm this discovery, a range of concentration from 10 to 70 µg ml^−1^ of 20 nm PS‐NPs were tested to assess their dose‐dependent on *Arabidopsis* growth. We found that the inhibitory effects on root growth were directly proportional to the concentrations of 20 nm PS‐NPs in the growth medium (Figure S2a, Supporting Information). For example, while high concentrations (> 60 µg ml^−1^) of 20 nm PS‐NPs nearly completely inhibited root development (Figure 1h; Figure S2b,c, Supporting Information), a concentration of 40 µg ml^−1^ only partially suppressed root growth (Figure 1i,j). In contrast, similar concentration variations of larger PS‐NPs (100, 200, 500, and 1000 nm) did not significantly affect root growth (Figure S3, Supporting Information). These results underscore the critical role of size and dosage of PS‐NPs in determining their phytotoxicity.

Given the cellulose matrix in the plant cell wall, the uptake of nanoparticles is largely governed by their size.^[^ ^50^ ^]^ Nanoparticles of smaller sizes are more readily taken up by plant roots.^[^ ^51^ ^]^ Our SEM images further support this, showing that 20 nm PS‐NPs, being the smallest size tested, are efficiently internalized by plant root tissues (Figure S4, Supporting Information).^[^ ^25^, ^27^, ^52^ ^]^ Generally, small‐sized nanoplastics are more likely to penetrate plant tissues.^[^ ^53^ ^]^ However, larger‐sized particles tend to distort and deform cell walls, leading to larger pores, thus allowing larger particles to enter the plant.^[^ ^54^ ^]^ Previous studies also have been reported to affect the growth of various plant species, including wheat, rice, corn, cucumbers and lettuce.^[^ ^55^, ^56^ ^]^ In summary, our results indicate that the growth defects caused by larger PS‐NPs are less severe than those cause by smaller PS‐NPs, suggesting distinct toxicity mechanism of small‐sized ones.

### 2.2 The Small PS‐NPs Disrupt *Arabidopsis* Root Development by Damaging Cell Viability and Cell Division Activity in Root Meristem

To explore the mechanisms underlying the toxicity of small PS‐NPs on root development, we investigated their effect on the primary root, which has a complex structure consisting of functionally distinct cell types.^[^ ^57^ ^]^ The primary root is divided into three distinct zones: the meristem zone, characterized by active cell division; the elongation zone, where cells divide slowly but elongate rapidly; and the maturation zone, comprising differentiated, non‐dividing cells.^[^ ^58^ ^]^ Propidium iodide (PI), a fluorescent dye, is able to penetrate the membranes of dead or damaged cells, binds to cellular DNA and RNA, enabling visualization of cell damage.

**Figure 2:**
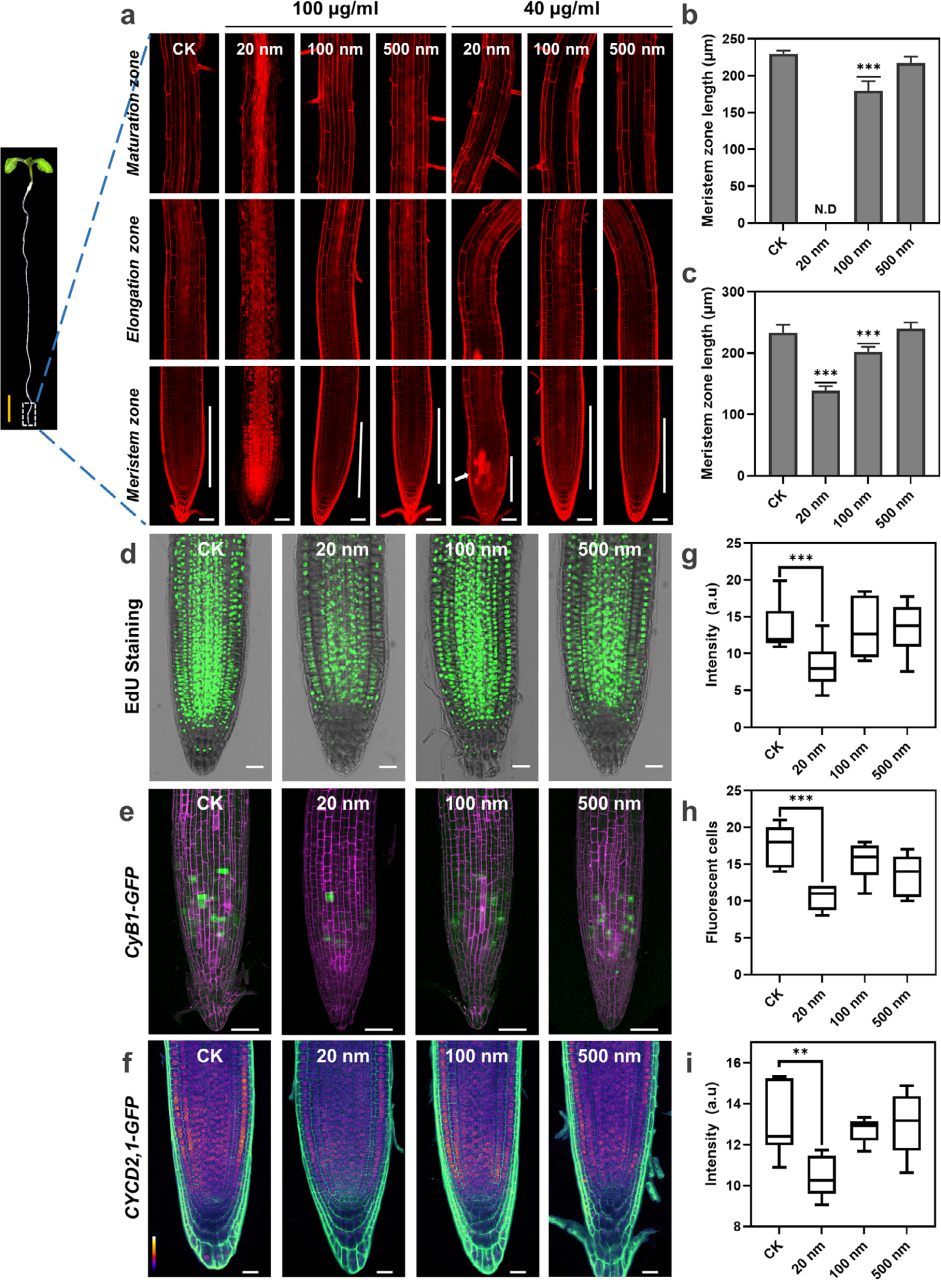
The small PS‐NPs damage the root meristem zone, including disruption of its cell viability and inhibition of cell division activity, to impair *Arabidopsis* root development. a) Representative images of 4‐day‐old seedling root tips stained with 5% propidium iodide (PI) to highlight the meristem zone, elongation zone and maturation zone after 2 days of incubation with 100 or 40 µg ml^−1^ 20, 100 or 500 nm PS‐NPs. White lines indicate the size of the proximal meristem region, except for the case treated with 100 µg ml^−1^ 20 nm PS‐NPs, which caused severe damage to root cells. Yellow and white scale bar: 0.2 cm and 50 µm, respectively. b,c) Measurement of root meristem zone following the treatment with 100 µg ml^−1^ (b) or 40 µg ml^−1^ (c) PS‐NPs, respectively. d) EdU staining to illustrate cell division activity in *Arabidopsis* root apical meristem treated with 40 µg ml^−1^ PS‐NPs for 24 h. Scale bar, 25 µm. e) Root meristems of *CyB1‐GFP* plants, showing a reduced number of cells expressing the marker after treatment with 20 nm PS‐NPs for 24 h. Scale bar, 50 µm. f) Confocal images of *CYCD2,1‐GFP* transgenic plants after 24 h of treatment with different sizes of PS‐NPs. The GFP channel images are displayed in pseudo color with the intensity scale shown at the lower left (White and black indicate high and low, respectively). Scale bar, 25 µm. g) Quantification of cells stained by EdU staining in (d). h) Quantification of cells expressing CyB1‐GFP from the experiment depicted in (e). i) Quantification of the fluorescence intensity of CYCD2,1‐GFP from the experiment depicted in (f). The boxplots represent the first to the third quartiles of the data, and whiskers indicate the minimum and maximum values. The line in the box indicates the mean value (g, h, i). The error bars represent the standard errors. Statistical analysis was performed using one‐way ANOVA followed by Dunnett's multiple comparisons test. * *p* < 0.05, **P < 0.01, *** *P* < 0.001, N.D, Not Detected.

Using PI staining, we observed extensive cell damage across all root zones ‐ including meristem, elongation and maturation zones ‐ in plants exposed to 100 µg ml^−1^ of 20 nm PS‐NPs for 48 h (**Figure** **2**a; Figure S5a, Supporting Information). This damage was indicated by intense red fluorescence under confocal microscopy, suggesting severe injury to root cells. Further analysis with a lower concentration (40 µg ml^−1^) of 20 nm PS‐NPs revealed that the root meristem zone was significantly shortened, with red fluorescence spots visible in this region, indicating cellular damage (Figure 2a; Figure S5b, Supporting Information). In contrast, the root elongation and maturation zones appeared relatively unaffected by 20 nm PS‐NPs at this low concentration. These results suggest that the meristem zone is particularly vulnerable to even low concentrations of small PS‐NPs, leading to damage in the meristem and, consequently, stunted root growth. Larger PS‐NPs (100, 200, 500, and 1000 nm) showed no significant effects on any root zones, regardless of whether they were administered at high (100 µg ml^−1^) or low (40 µg ml^−1^) concentrations (Figure 2a–c; Figures S5 and S6, Supporting Information).

Additionally, considering that the plant root system includes both primary and lateral roots,^[^ ^59^ ^]^ we extended our analysis to examine lateral root development under various PS‐NPs treatments. Phenotypic analysis revealed that smaller PS‐NPs, particularly those sized 20 nm, significantly disrupted lateral root growth, resulting in substantially shorter lateral roots compared to untreated plants or those treated with larger PS‐NPs (100, 200, 500, and 1000 nm) (Figures S7 and S8, Supporting Information). PI staining revealed that the cells within the meristem and elongation zones of the lateral root were impaired after exposure to 100 µg ml^−1^ of 20 nm PS‐NPs treatment, similar to the effects observed in the primary roots, thereby impeding their growth (Figure 2a; Figure S7, Supporting Information). Remarkably, lateral roots appeared less sensitive to small PS‐NPs, as in contrast to the primary root maturation zone, no significant damage was observed after exposure to 20 nm PS‐NPs at 100 µg ml^−1^. Additionally, PI staining of lateral roots treated with larger PS‐NPs (100, 200, 500, and 1000 nm) showed no red fluorescence within the cells, similar to untreated roots, indicating no severe damage to cells.

At high concentrations, 20 nm PS‐NPs rapidly compromised cell integrity, thereby inhibiting root growth instantly and preventing the examination of their effect on root growth at the genetic level. Consequently, low concentration (40 µg ml^−1^) was employed to investigate the impact of these nanoplastics on gene expression regulation and cellular signal pathways in greater detail. The root apical meristem (RAM) is the primary tissue involved in active cell division, which is crucial for continuous cell replenishment and maintenance of root growth. To assess the effect of PS‐NPs on RAM cell division activity, we performed a 5‐ethynyl‐2′‐deoxyuridine (EdU) incorporation assay, which is commonly used to indicate the active cell division in the DNA synthesis (S phase).^[^ ^60^, ^61^, ^62^ ^]^ We found that EdU incorporation in plants treated with 20 nm PS‐NPs was much slower than the untreated plants or those treated with larger PS‐NPs, suggesting that these small nanoplastics strongly inhibited cell division in the meristem (Figure 2d,g; Figure S9, Supporting Information). We utilized transgenic *Arabidopsis* lines expressing two cell division markers, *CyB1 ‐ GFP* (*cyclin B‐green fluorescent protein*) and *CYCD2,1‐GFP*, which respectively reflect the G2‐to‐M phase transition and G1‐to‐S phase transition in the cell cycle, to identify the cell mitotic activity.^[^ ^63^, ^64^ ^]^ The *CyB1‐GFP* indicated that the PS‐NPs sized 20 nm significantly inhibited cell division activity (Figure 2e,h; Figure S10a, Supporting Information), and the *CYCD2,1‐GFP* further confirmed this conclusion (Figure 2f,i; Figure S10b, Supporting Information). On the contrary, roots treated with larger PS‐NPs (100, 200, 500, and 1000 nm) showed *CyB1‐GFP* and *CYCD2,1‐GFP* signals comparable to those in untreated roots (Figure S10, Supporting Information). These findings suggest that small PS‐NPs, rather than larger ones, inhibit cell division in RAM, which likely accounts for the observed phenotype of shortened root meristem zones and impaired root growth following exposure to 20 nm PS‐NPs.

In summary, small PS‐NPs exhibit significantly greater phytotoxicity than larger ones in both primary and lateral root development. Exposure to small PS‐NPs results in cellular damage and a reduction in cell division activity in the root meristem, which is critical for maintaining root growth. This also underscores the heightened sensitivity of the RAM to small nanoplastics.

### 2.3 Activation of Gene Expression Related to Root Meristem Homeostasis Maintenance to Combat Phytotoxicity of Small Nanoplastics

**Figure 3:**
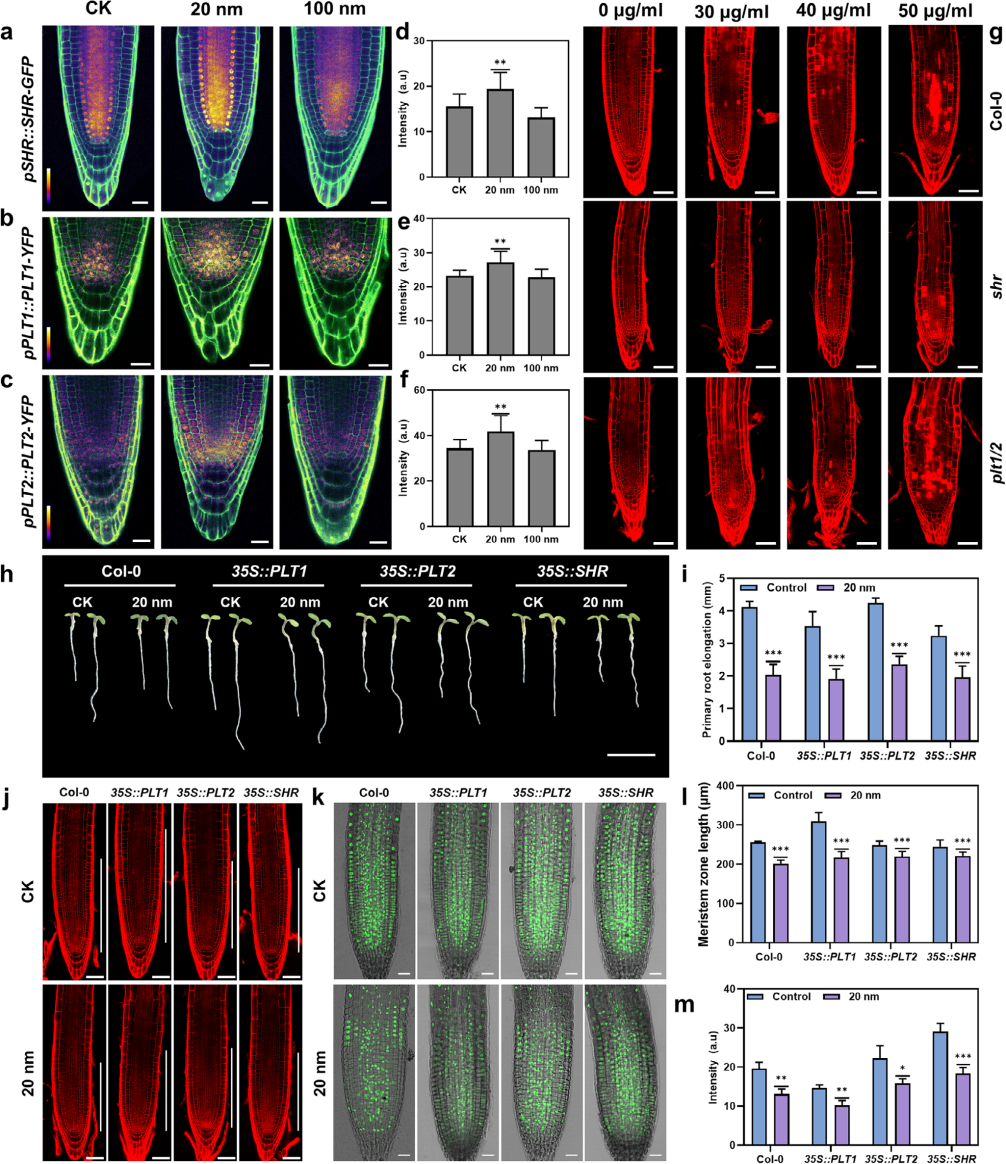
Root response to phytotoxicity induced by small PS‐NPs through activation of gene expression related to meristem maintenance and wound healing. a–c) Expression patterns of *pSHR::SHR‐GFP* (a), *pPLT1::PLT1‐YFP* (b), and *pPLT2::PLT2‐YFP* (c) in root tips of transgenic seedlings without treatment (CK) and treated with 40 µg ml^−1^ of 20 and 100 nm PS‐NPs for 24 h. GFP channel images are presented in pseudo color, with the intensity scale shown at the lower left (White and black indicate high and low, respectively). Scale bar, 20 µm. d–f) Quantitative analysis of GFP signal intensity in *pSHR::SHR‐GFP* (d), *pPLT1::PLT1‐YFP* (e), and *pPLT2::PLT2‐YFP* (f) transgenic seedlings shown in (a‐c). g) Representative images of PI‐stained roots from different mutant lines (*plt1/2* and *shr*) treated with varying concentrations of 20 nm PS‐NPs, which showed increased cell damage by small PS‐NPs compared to the wild type. Scale bar, 50 µm. h,i) Phenotypic analysis (h) and quantification (i) of primary root elongation in overexpression lines *35S::PLT1*, *35S::PLT2*, and *35::SHR* treated with 40 µg ml^−1^ 20 nm PS‐NPs for 24 h. Scale bar, 0.5 cm. j,k) PI staining (j) and EdU staining (k) in root tips of *35S::PLT1*, *35S::PLT2*, and *35::SHR* following exposure to 40 µg ml^−1^ 20 nm PS‐NPs for 24 h. Scale bar, 50 µm for (j) and 25 µm for (k). l,m) Measurements and quantification of meristem zone length shown in (j) and fluorescence signal intensity shown in (k), respectively. Data are presented as mean ± standard error (SE). Statistical analysis was conducted using one‐way ANOVA followed by Dunnett's multiple comparisons test. * *p* < 0.05, **P < 0.01, *** *P* < 0.001.

Plants have evolved regenerative capacities to repair damaged tissues in response to prolonged exposure to biotic or abiotic stresses.^[^ ^65^, ^66^ ^]^ Two parallel signaling pathways, mediated by PLETHORA (PLTs) proteins and SHORTROOT‐SCARECROW (SHR/SCR) pathways, are known to be critical for maintaining RAM homeostasis.^[^ ^67^, ^68^ ^]^ To explore the role of these pathways in plant responses to the phytotoxicity of small nanoplastics, we used the transgenic *Arabidopsis* lines, *pSHR::SHR‐GFP*, *pPLT1::PLT1‐YFP*, and *pPLT2::PLT2‐YFP*. Treatment with 40 µg ml^−1^ of 20 nm PS‐NPs significantly upregulated the expression of *SHR*, *PLT1*, and *PLT2* genes (**Figure** **3**a–f). In contrast, exposure to larger PS‐NPs (100, 200, 500, and 1000 nm) did not significantly alter the expression of these genes (Figures S11 and S12, Supporting Information).

To confirm the critical role of *SHR* and *PLT* genes in plant resistance to phytotoxicity of small PS‐NPs, we obtained *shr* single mutant and *plt1/plt2* double mutant. The *plt1/plt2* mutant exhibits more extensive damage to the root when exposed to increasing concentrations of 20 nm PS‐NPs (from 30 to 50 µg ml^−1^) compared to the wild type. Similarly, the *shr* mutant showed greater sensitivity to 20 nm PS‐NPs than the wild type (Figure 3g). These results suggest that both *SHR* and *PLT* genes play a positive role in enhancing plant resistant to small PS‐NPs.

To further validate this, we overexpressed *SHR*, *PLT1*, and *PLT2* genes in the wild type to examine their roles in plant responses to small PS‐NPs. We found that compared to the wild type, which exhibited a 50.51 ± 5.38% inhibition in primary root length after exposure to 20 nm PS‐NPs for 24 h, overexpression lines *35S::PLT1*, *35S::PLT2*, and *35S::SHR* displayed reduced growth inhibition, with values of 45.9 ± 3.86%, 43.97 ± 2.71%, and 38 ± 7%, respectively (Figure 3h,i). Concurrently, PI staining indicated that the RAMs in these overexpressing lines were much longer than in the wild type after exposure to 20 nm PS‐NP (Figure 3j,l). EdU staining further demonstrated that cell division activity in the RAMs of *35S::PLT1*, *35S::PLT2*, and *35S::SHR* lines was much higher than in the wild type following treatment with 20 nm PS‐NP (Figure 3k,m). These results strongly suggest that the up‐regulation of *PLT1*, *PLT2*, and *SHR* genes in response to small PS‐NPs serves as an adaptive strategy, enabling plants to combat the phytotoxicity of tiny nanoplastic particles in root growth. This highlights the essential role of PLT‐ and SHR/SCR‐mediated signaling pathways in maintaining RAM homeostasis under plants are exposed to small nanoplastics.

### 2.4 Transcriptome Analysis Reveals Alterations in Auxin Signaling Pathways in Roots Exposed to Small PS‐NPs

**Figure 4:**
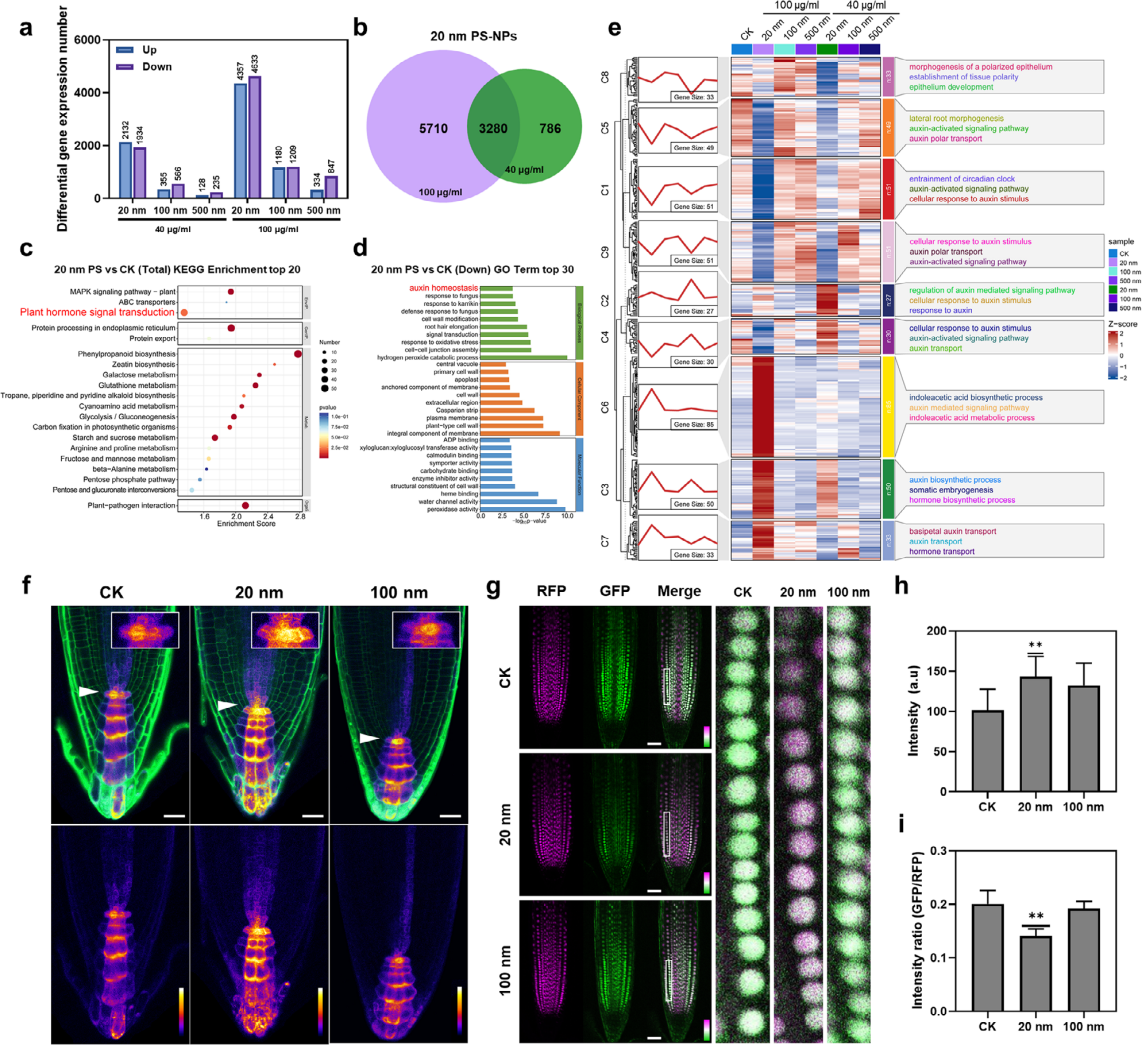
Small PS‐NPs, rather than larger PS‐NPs, inducing auxin accumulation in roots. a) Transcriptomic analysis identified the differentially expressed genes (DEGs) in *Arabidopsis* roots exposed to 100 µg mL^−1^ or 40 µg mL^−1^ of 20, 100, 500 nm PS‐NPs for 24 h, compared to those without treatment. b) Venn diagrams of overlapping DEGs in roots treated with 100 µg mL^−1^ and 40 µg mL^−1^ 20 nm PS‐NPs for 24 h. c) Kyoto Encyclopedia of Genes and Genomes (KEGG) pathway enrichment analysis of overlapping DEGs shown in (b). Circles represent the number of genes encoded by differential proteins, with color indicating *p*‐value levels (Red: Low, Blue: High). d) Gene Ontology (GO) terms enrichment analysis of down‐regulated DEGs of *Arabidopsis* root under exposure to 100 and 40 µg ml^−1^ 20 nm PS‐NPs. e) Detailed Temporal Analysis of DEGs cluster expression patterns (C1‐C9) in response to 100 µg ml^−1^ or 40 µg ml^−1^ PS‐NPs in *Arabidopsis* roots. Red lines in left panels depict expression trends of associated DEGs, with the number labeled below the frame. Diagrams on the right illustrate functional enrichment of genes for the nine clusters, with red indicating upregulation and blue indicating downregulation. f) Auxin‐responsive marker line *DR5rev::GFP* illustrating the enhanced auxin response in roots exposure to 40 µg ml^−1^ 20 nm PS‐NPs compared to the non‐treated roots. GFP channel images are displayed in pseudo color, with intensity scale shown at the lower right (White and black indicate high and low, respectively). White arrows point to the quiescent center. Scale bar: 20 µm. g) The *R2D2*, as a semiquantitative and rapid auxin‐input reporter, indicating the enhanced auxin response in roots treated with 40 µg ml^−1^ 20 nm PS‐NPs relative to non‐treated roots. Scale bar 50 µm. h,i) Quantification of the GFP intensity of *DR5rev::GFP* shown in (f) and *R2D2* shown in (g). Error bars represent the standard deviations. Statistical analysis was performed using one‐way ANOVA followed by Dunnett's multiple comparisons test. * *p* < 0.05, **P < 0.01, *** *P* < 0.001.

To elucidate the molecular mechanisms underlying the effects of PS‐NPs on *Arabidopsis* root growth, we conducted RNA sequencing of root samples treated with different sizes of PS‐NPs for transcriptomic analysis. Correlation and principal component analyses revealed significant differences in gene expression between roots treated with different PS‐NPs and untreated control (Figure S13, Supporting Information). As expected, high concentration (100 µg ml^−1^) of both small and large PS‐NPs resulted in a greater number of differentially expressed genes (DEGs) compared to lower concentration (40 µg ml^−1^). Notably, roots exposed to 20 nm PS‐NPs exhibited a significantly higher number of DEGs compared to those treated with 100 or 500 nm PS‐NPs, indicating a more profound effect of small PS‐NPs on gene expression (**Figure** **4**a).

Further analysis identified 3280 DEGs regulated by both 40 and 100 µg ml^−1^ 20 nm PS‐NPs, suggesting these genes are strongly associated with plant response to 20 nm PS‐NPs (Figure 4b). Functional annotation using the Kyoto Encyclopedia of Genes (KEGG) analysis revealed that pathways such as plant hormone signal transduction, phenylpropanoid biosynthesis, and MAPK signaling pathways, were significantly affected by exposure to 20 nm PS‐NPs (Figure 4c; Figure S14, Supporting Information). Phenylpropane biosynthesis is the main pathway for the synthesis of flavonoid and lignin. Previous studies reported that changing the expression of genes related to this pathway, thereby regulating enzyme activity and metabolite accumulation, is an important way for plant to cope with abiotic stresses.^[^ ^69^ ^]^ In addition, zeatin, as a phytohormone, controls the process of cell division and regulates the formation of root.^[^ ^70^ ^]^ Plants achieve the decomposition of sugars through the TCA cycle to obtain the energy.^[^ ^71^ ^]^ KEGG enrichment results show that small nanoplastics affected the cell division of root, stimulated the starch and sucrose metabolism and produced more energy to maintain its growth. Gene Ontology (GO) annotation further confirmed that the auxin homeostasis pathway was down‐regulated in roots exposed to 20 nm PS‐NPs (Figure 4d; Figure S15, Supporting Information). Simultaneously, GO enrichment also finds that small nanoplastics also affected the cell‐cell junction assembly and integral component membrane pathways. Nanoplastics enter plant roots accumulate in the extracellular space or cell wall layer, where they interact with plasma membrane.^[^ ^72^ ^]^ This interaction triggers excessive production of reactive oxygen species, disrupts the cell recycle, and ultimately leads to the destruction of plant cell structure.^[^ ^73^ ^]^

The small PS‐NPs also induced alterations in phenylpropanoid biosynthesis and MAPK signaling pathways, both of which are crucial for maintaining auxin homeostasis.^[^ ^74^ ^]^ Previous studies have shown that flavonoids, secondary metabolites of phenylpropanoid biosynthesis, can interfere with the activity of auxin efflux transporter PIN‐FORMED (PIN) proteins, thereby affecting auxin transport.^[^ ^75^ ^]^ Short‐Time Sequence Expression Mining (STEM) analysis of GO‐annotated DEGs related to phytohormones categorized them into nine clusters (Figure 4e). As the concentration of 20 nm PS‐NPs increased, the biosynthesis of phytohormones (e.g., indoleacetic acid) and the auxin‐mediated signaling pathway in *Arabidopsis* roots were enhanced, while auxin transport was diminished. This likely explains the observed defects in lateral root development (Figure S8, Supporting Information). In contrast, roots treated with either high or low concentrations of larger PS‐NPs (100 and 500 nm) showed no significant changes in DEGs compared to untreated roots.

Polar auxin transport (PAT) within the root establishes an auxin gradient at the root apex, termed the auxin maximum, which is essential for maintaining the stem cell niche in root meristem for cell division and differentiation.^[^ ^76^ ^]^ To further validate the transcriptomic results, we introduced transgenic lines expressing the auxin responsive reporter *DR5rev::GFP* or *R2D2*. In response to 20 nm PS‐NPs, the *DR5rev::GFP* signal was significantly enhanced (Figure 4f,h). However, in roots treated with larger PS‐NPs (100, 200, 500, and 1000 nm), the *DR5rev::GFP* signal was similar to that in untreated *Arabidopsis* roots (Figure S16a, Supporting Information). Additionally, the *R2D2* reporter, which provides a semi‐quantitative measure of auxin accumulation, showed that the DII‐VENUS signal was dramatically reduced in roots exposed to 20 nm PS‐NPs compared to untreated roots or those treated with larger PS‐NPs (100, 200, 500, and 1000 nm). The mDII‐ntdTomato signal, serving as an internal control, remained constant across all treatments (Figure 4g,i; Figure S16b, Supporting Information). These results were further verified by the transgenic line *DII::VENUS* that higher concentrations of auxin accumulation caused more pronounced protein degradation, especially treated with 20 nm PS‐NPs (Figure S17, Supporting Information).

In summary, small PS‐NPs significantly alter gene expression at a larger‐scale than their larger counterparts, disrupting cellular signaling pathways, particularly auxin signal transduction. This is consistent with our findings from the auxin reporters *DR5rev::GFP* and *R2D2*, which revealed enhanced endogenous auxin accumulation in root tips following exposure to 20 nm PS‐NPs.

### 2.5 Small PS‐NPs Disrupt the Expression and Apical Localization of the Auxin Efflux Transporter PIN2 in Root Epidermal Cells

**Figure 5:**
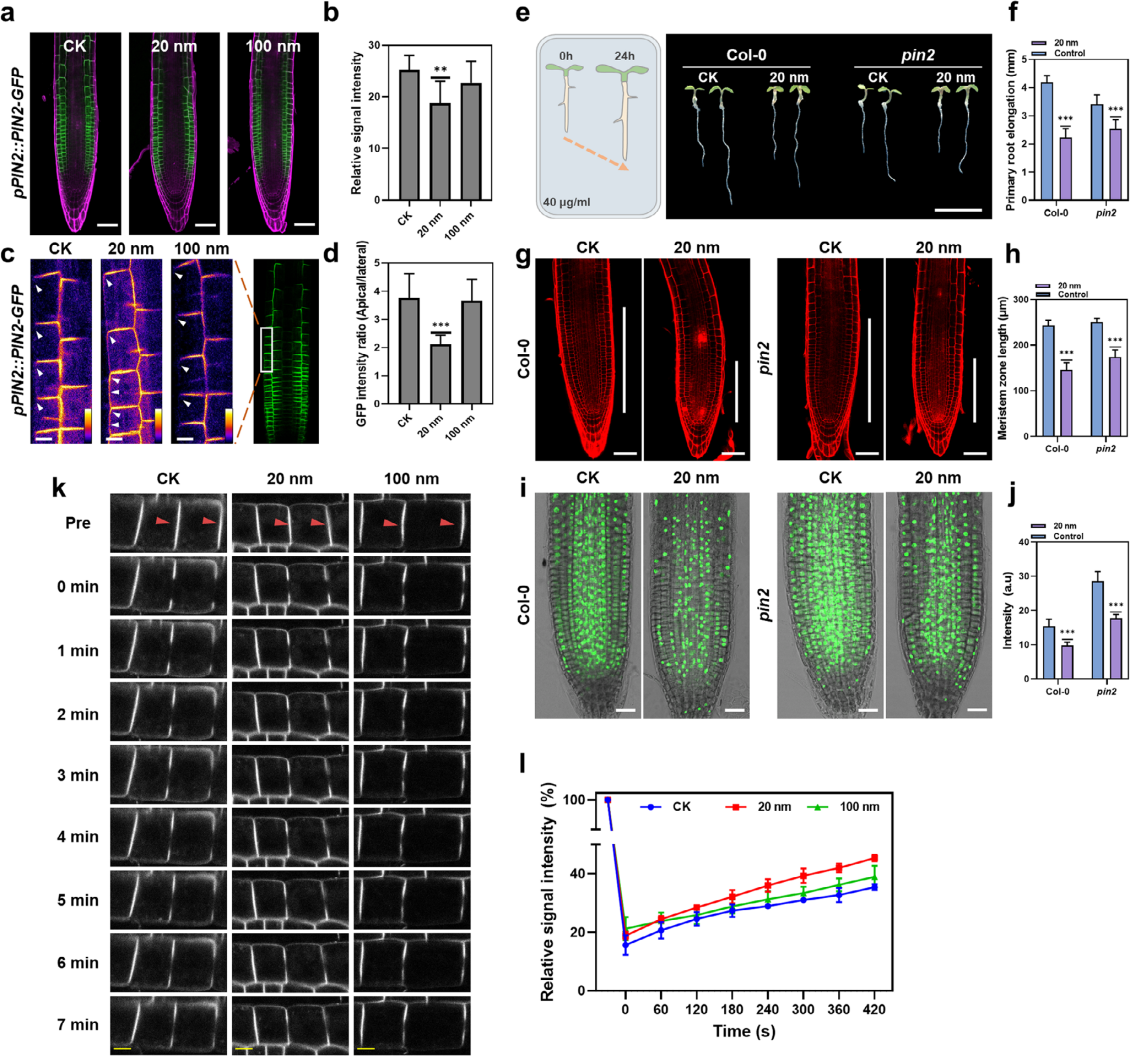
In response to small PS‐NPs, the function of auxin efflux transporter PIN2 is attenuated to accumulate auxin in root tips for plant resistance to its phytotoxicity. a) The *pPIN2::PIN2‐GFP* transgenic lines showing the downregulation of PIN2 in roots treated with 40 µg ml^−1^ 20 nm PS‐NPs compared to non‐treated roots and those treated with 100 nm PS‐NPs. Scale bar 50 µm. b) Quantification of PIN2‐GFP signal intensity shown in (a). c) PIN2 polarity at the plasma membrane of epidermal cells without treatment or with 40 µg ml^−1^ PS‐NPs treatment. White arrows indicate the PIN2‐GFP signal at the lateral side of epidermal cells, demonstrating the decreased PIN2 polarity following the exposure to 20 nm PS‐NPs treatment. GFP signal intensity represented using pseudo‐color (white indicates high intensity, black indicates low intensity). Scale bar 10 µm. d) Quantitative analysis of the PIN2‐GFP signal between apical and lateral sides of root epidermal cells. e) Phenotypic comparison of root growth between wild type (Col‐0) and *pin2* mutant without treatment or after treatment with 40 µg ml^−1^ 20 nm PS‐NPs for 24h. Scale bar, 1 cm. f) Measurement of primary root elongation shown in (e). g) PI‐stained roots of wild type and *pin2* mutant exposed to 40 µg ml^−1^ 20 nm PS‐NPs for 24 h. Scale bar, 50 µm. h) Quantification of root meristem sizes shown in (g). i) EdU staining of roots in Col‐0 and *pin2* mutant without or with 40 µg ml^−1^ 20 nm PS‐NPs treatments. Scale bar, 25 µm. j) Quantification of EdU fluorescence intensity shown in (i). k) Fluorescence recovery after photobleaching (FRAP) dynamics of PIN2‐GFP in root epidermal cells without treatment or 40 µg ml^−1^ PS‐NPs treatment for 24 h. Scale bar: 5 µm. l) Quantitative analysis of the fluorescence recovery rate of PIN2‐GFP after photobleaching in (k). Statistical analysis was conducted using one‐way ANOVA followed by Dunnett's multiple comparisons test. * *p* < 0.05, **P < 0.01, *** *P* < 0.001.

Transcriptomic analysis indicated that small PS‐NPs affect the auxin transport pathway. Directional auxin flow relies on auxin transporters, such as auxin efflux carrier PIN proteins and influx carrier AUX1/LIKE‐AUX1 (AUX/LAX) proteins.^[^ ^77^, ^78^, ^79^ ^]^ PAT establishes an auxin gradient at the root apex, which is essential for root development and tropic growth. To assess the differential expressions of auxin transporter genes in response to different sizes of PS‐NPs, we used *Arabidopsis* transgenic lines *pPIN1::PIN1‐GFP*, *pPIN2::PIN2‐GFP*, *pPIN3::PIN3‐GFP*, *pPIN7::PIN7‐GFP*, and *pAUX1::AUX1‐YFP*. The results showed that PIN2‐GFP and AUX1‐YFP signals, primarily expressed in the outmost cells of the roots, were significantly downregulated following exposure to 20 nm PS‐NPs (**Figure** **5**a,b; Figures S18b and S19, Supporting Information). In contrast, the signals of PIN1‐GFP, PIN3‐GFP, and PIN7‐GFP were only slightly or not affected (Figure S18a,c,d,e, Supporting Information). These findings suggest that small PS‐NPs primarily influence the expression levels of auxin transporters localized in root epidermal cells like PIN2 and AUX1. PIN2, predominantly localized at apical side of the plasma membrane (PM) in root epidermal cells, plays a critical role in upward auxin transport. Therefore, the downregulation of PIN2 following after exposure to 20 nm PS‐NPs inhibits the upward flow of auxin, resulting in auxin accumulation at the root apex (Figure 4f,g).

Beyond expression levels, the apical localization of PIN proteins in the PM is crucial for their function in directing auxin flow.^[^ ^80^ ^]^ We next investigated whether small PS‐NPs alter the polarity of PIN2 localization in epidermal cells. Our results showed that exposure to 20 nm PS‐NPs significantly disrupted this polarity, with a strong PIN2‐GFP signal detected at the lateral side of epidermal cells (Figure 5c,d). In roots treated with larger PS‐NPs (100, 200, 500, and 1000 nm), the PIN2‐GFP signal remained comparable to untreated roots, with no GFP signal observed at the lateral side of epidermal cells (Figure S20, Supporting Information).

To explore the mechanism behind the change of PIN2 polarity in the PM, we examined the lateral diffusion rate of the PIN2 protein within the PM. Its lateral diffusion rate is closely associated with the establishment and maintenance of protein polarity.^[^ ^81^ ^]^ We performed Fluorescence Recovery After Photobleaching (FRAP) assay to evaluate the PIN2 lateral diffusion rate. Our results revealed that, following exposure to 20 nm PS‐NPs, the recovery rate of PIN2‐GFP in the PM was significantly faster compared to untreated cells. This may be because small nanoplastics with high specific surface areas are more likely to interact with PM and penetrate into the hydrophobic core of the bilayer lipid membrane. At same time, the oxidative stress induced by nanoplastics exposure also oxidizes components in the phospholipid bilayer, which causes changes in cell membrane structure and functional damage.^[^ ^38^, ^82^ ^]^ In contrast, the recovery rate of PIN2‐GFP in roots treated with larger PS‐NPs (100 nm) showed no difference from untreated cells (Figure 5k,l). These findings suggest that small PS‐NPs enhance the lateral diffusion rate of PIN2 within the plasma membrane, thereby diminishing PIN2 polarity in epidermal cells. In contrast, larger PS‐NPs do not have this effect.

Taken together, these results indicate that small nanoplastic particles, unlike the large counterparts, can reduce PIN2 expression and disrupt its apical polarity in root epidermal cells by enhancing its lateral diffusion rate within the PM. This alteration in PIN2 function impedes the normal auxin flow from the root apex to elongation zone, resulting in auxin accumulation in the root apex.

### 2.6 PIN2‐Dependent Auxin Accumulation in Roots Confers Plant Resistance to PS‐NPs

Auxin plays a critical role in plant response to abiotic stress.^[^ ^83^ ^]^ To determine whether the inhibition of PIN2 function induced by 20 nm PS‐NPs leads to increase auxin accumulation in roots as an adaptive strategy for plant growth, we use the loss‐of‐function *pin2* mutant to examine its response to small PS‐NPs. The results confirmed that *pin2* mutant displayed significantly stronger resistance to the detrimental effects of small PS‐NPs compared to the wild type. Specifically, the inhibitory effect of 20 nm PS‐NPs on root growth was less pronounced in the *pin2* mutant than in the wild type. And compared with nearly half inhibition of wild type, the *pin2* mutant displayed attenuated inhibitory response to small nanoplastics as evidenced by 25 ± 5% reduction in primary root elongation (from 3.41 ± 0.33 to 2.54 ± 0.32 mm). (Figure 5e,f).

Additionally, upon exposure to 20 nm PS‐NPs, PI staining indicated that the *pin2* mutant exhibited significantly longer root meristem zones and less cell damage in the meristem compared to the wild type (Figure 5g,h). EdU incorporation assays further demonstrated that cell division activity in the meristem of the *pin2* mutant was better maintained than in the wild type due to the accumulation of auxin caused by impaired upward transport^[^ ^84^ ^]^ (Figure 5i,j). These findings confirm that the inhibition of PIN2 function, which enhances the auxin accumulation in the roots, enabling plants to better withstand the phytotoxicity of small nanoplastic particles.

### 2.7 Small PS‐NPs Disrupt Asymmetric Auxin Flow to Inhibit Root Gravitropic Growth

**Figure 6:**
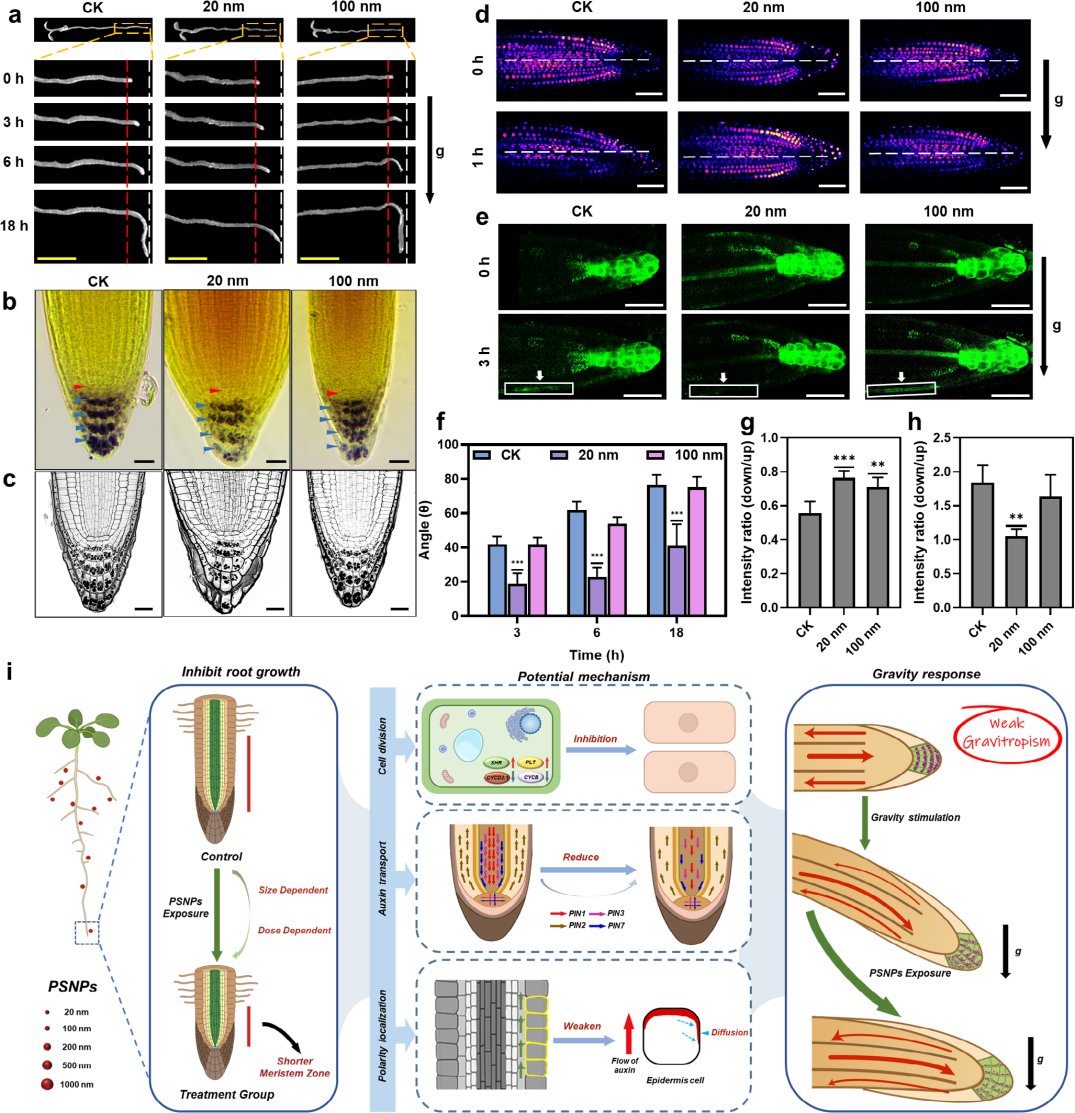
Small PS‐NPs inhibit the auxin redistributions to impair the adaptive root growth ‐ gravitropism. a) Phenotypic analysis of root gravitropism of *Arabidopsis* roots with no treatment (CK) or 40 µg ml^−1^ PS‐NPs treatment. Roots were gravistimulated for various time intervals following a 90° reorientation. Scale bars: 0.2 cm. b,c) Lugol's staining (b) and mPS‐PI staining (c) analysis of starch granule accumulation in *Arabidopsis* root apex treated with 40 µg ml^−1^ PS‐NPs for 24 h. Red arrowheads indicate the quiescent center (QC) position. Scale bars: 50 µm for (b), 20 µm for (c). d,e) The auxin redistribution in roots with no treatment (CK) or 40 µg ml^−1^ PS‐NPs treatment upon gravistimulation, which was indicated by the auxin‐responsive reporter *DII::VENUS* (d) and *DR5rev::GFP* (e). The black arrows indicated the direction of gravity stimulation. Scale bar 50 µm for (d), 100 µm for (e). f) Quantification of root bending curvatures shown in (a). g,h) Quantification of fluorescence signal intensity ratio between upper and lower root in (d, e). i) Schematic diagram representing the phytotoxicity of small PS‐NPs (20 nm) on *Arabidopsis* roots and the difference of plant gravitropic growth dynamic that are induced in response to small PS‐NPs. Data are presented as mean ± standard error (SE). Statistical significance was assessed using one‐way ANOVA followed by Dunnett's multiple comparisons test. * *p* < 0.05, **P < 0.01, *** *P* < 0.001.

Root gravitropism is essential for shaping root system architecture (RSA) in soil, enabling plant to adapt to environmental changes. Since direction auxin flow is essential for root tropic growth, we investigated the effect of PS‐NPs on *Arabidopsis* root gravitropism. As shown in **Figure** **6**a,f, treatment with 20 nm PS‐NPs inhibited root gravitropism, resulting in significantly decreased bending curvature after gravistimulation compared to untreated roots or those treated with larger PS‐NPs (100, 200, 500, and 1000 nm) (Figure S21, Supporting Information).

To explore the mechanism underlying the decreased root gravitropism induced by 20 nm PS‐NPs, we examined the root apex‐localized amyloplasts, which server as statoliths to sense gravity direction and mediate asymmetric auxin flow for root bending. Lugol's staining and mPS‐PI staining revealed that the content of amyloplasts in roots exposed to 20 nm PS‐NPs was reduced compared to untreated roots and those treated with larger PS‐NPs (Figure 6b,c; Figures S22 and S23, Supporting Information). This suggested that 20 nm nanoplastics affect the perception of gravity signals at the root tips in addition to polar transport of auxin. It may interfere with the expression of genes related to starch synthesis and degradation, and reduce the accumulation of starch granules in the root tips.^[^ ^85^ ^]^ Meanwhile, the small nanoplastics activated the antioxidant defense system in the plants, accelerating the metabolism of starch and sugars to maintain the energy required for growth.^[^ ^71^ ^]^ Additionally, exposure to 20 nm PS‐NPs disrupted asymmetric auxin redistribution following gravistimulation, as indicated by the strong, undegraded DII‐VENUS signal in the lower lateral part of the root (Figure 6d,g). In contrast, PS‐NPs of 100, 200, 500, and 1000 nm sizes had no effect on DII‐VENUS degradation in the lower part of the root following gravistimulation (Figure S24, Supporting Information). Further investigation with the *DR5rev::GFP* auxin reporter showed that, in untreated roots, auxin accumulated on the lower lateral side of the root after 3 h of gravistimulation, leading to asymmetric root growth and subsequent bending towards the gravity vector. However, in roots treated with 20 nm PS‐NP, the *DR5rev::GFP* signal was not clearly observed in the lower lateral part of the roots after gravistimulation (Figure 6e,h). In contrast, the signal was detected in roots treated with PS‐NPs of 100, 200, 500, and 1000 nm sizes, similar to untreated roots (Figure S25, Supporting Information).

Taken together, these results suggest that the small nanoplastics interfere with auxin flow to inhibit asymmetric redistribution of auxin along the root longitude axis, thereby impairing root gravitropic growth (Figure 6a). Mechanistically, this interference is likely due to a reduction in accumulation of root apex‐localized amyloplasts (Figure 6b,c), a decrease in PIN2 expression (Figure 5a,b), and the disruption of PIN2 polarity in the PM of root epidermal cells (Figure 5c,d).

## 3 Conclusion

Over the past century, global plastic production has surged, reaching a remarkable 367 million tons in 2020 alone.^[^ ^86^ ^]^ As a result, synthetic plastics are fragmented and degraded into micro‐ and nano‐scale particles within the environment, which raises critical concerns for human health and ecological sustainability.^[^ ^87^ ^]^ The accumulation of micro‐ and nano‐plastics within the food chain has become an important area of research, with a focus on their effects on plant health and ecosystems.^[^ ^88^ ^]^ Simultaneously, exogenous inputs from agricultural practices such as cultivation, fertilization, irrigation and mulching,^[^ ^89^, ^90^ ^]^ as well as long‐term environmental aging processes including light irradiation and climate warming to accelerates the degradation of polymeric materials likes plastics and rubber,^[^ ^91^ ^]^ leading to an increase in the production and accumulation of smaller nano‐plastic particles in soil.^[^ ^92^ ^]^ This introduces additional challenges for plant growth and environmental adaptation.

While previous studies have primarily focused on larger PS‐NPs, smaller particles, such as those sized at 20 nm, have received less attention despite their widespread presence in the environment.^[^ ^93^ ^]^ This study provides novel insights into the biological effects of nanoplastic particle smaller than 50 nm on plant development, shedding light on the underlying molecular mechanisms (Figure 6i). Our findings demonstrate that as the size of the nanoplastic particle decreases, their biological toxicity substantially increases. At the same concentration, 20 nm PS‐NPs dramatically compromised *Arabidopsis* root development and gravitropic growth, while larger PS‐NPs (100, 200, 500 and 1000 nm) exhibited minimal effects. We also elucidated the molecular mechanisms through which small PS‐NPs inhibit *Arabidopsis* root growth. Compared to larger PS‐NPs (≥ 100 nm), smaller PS‐NPs are much more likely to damage root cells, especially in the meristematic zones, which are crucial for root cell division and growth. Meanwhile, the damage caused by 20 nm PS‐NPs to *Arabidopsis* root meristem and maturation zones occurs in a concentration‐dependent manner, suggesting varying sensitivities to small nanoplastic particle across different plant tissues and organs. As a defensive response, plants activate two signaling pathways to enhance plant resistance to the biological toxicity of small PS‐NPs: i) upregulating the expression of *PLT1*, *PLT2*, and *SHR* genes associated with plant wound healing (Figure 3a–c), and ii) inhibiting the PIN2‐dependent upward auxin flow to accumulate auxin in roots (Figures 4f,g and 5a,b). The two pathways may be related to some extent. Specifically, local auxin accumulation in root may affect PLT expression through ARF‐dependent transcriptional activation, while PLT proteins may regulate auxin distribution by controlling auxin transport.^[^ ^94^, ^95^ ^]^ Both pathways jointly promoted plant resistance to small nanoplastic particles. However, this comes at the expense of root gravitropic growth, as PIN2‐mediated upward auxin flow is essential for root gravitropism. Therefore, the presence of small nanoplastic particles in the environment not only affects plant growth but also undermines the ability of plant to adapt to changing environmental conditions. There are many studies that leverage *Arabidopsis*‐generated knowledge to greatly help us improve adaptability of crops to various environmental stresses and increase the production and quality of agricultural practice.^[^ ^96^ ^]^ This study provides new insights into understanding the adaptive strategies adopted by plants to cope with nanoplastics stress, but in‐depth assessments of impacts of nanoplastics on other plants, especially crops, still need to consider differences in physiological metabolism, genetic background, etc.^[^ ^97^ ^]^ As a potential challenge in agricultural production, it is crucial to address how to mitigate the uptake of micro‐ and nanoplastics by plants and their adverse effects on abiotic stresses, particularly in crops. These findings offer significant theoretical support for the scientific evaluation of nanoplastics phytotoxicity and the development of integrated risk mitigation measures.

Sustainable agriculture has largely focused on increasing productivity to meet growing global demands. However, the pressure of a rising populations has prompted the widespread use of artificial measures, such as mulching, which, coupled with environmental pollution, excessive fertilization, and other detrimental practices, has led to a significant decline in the quality of arable land.^[^ ^98^ ^]^ The environmental risks posed by nanoplastics are becoming increasingly evident. Our study lays the foundation for further assessments of these materials. We demonstrated that the uptake and internalization of nanoplastics by plant roots can impair normal growth and weaken the plant response to surrounding environmental factors, ultimately reducing nutrient accessibility. Agricultural production is an important guarantee for global food security. Chronic exposure to nanoplastics will not only cause phytotoxic effects, threatening the stability of crop yields and nutritional quality, but may also be enriched in agricultural products through the food chain, ultimately posing a potential risk to human health. Addressing the challenges posed by micro‐ and nanoplastics in agricultural production is essential. Strategies to accelerate the degradation of these pollutants in the environment and mitigate their uptake by plants ‐ especially crops ‐ should receive significant attention from researchers, policymakers, and agricultural practitioners alike.

## 4 Experimental Section

### Reagents and Plant Materials

PS‐NPs of varying sizes were custom synthesized by Duke Scientific Corporation (USA) and supplied as a suspension in ultrapure water. The nominal particle sizes of PS‐NPs were 20, 100, 200, 500, and 1000 nm, with actual sizes characterized via Scanning Electron Microscope (SEM) and Transmission Electron Microscopy (TEM). Details of methods are listed in Text S1‐S2 (Supporting Information). Deionized water was used throughout the experiment.

*Arabidopsis thaliana* with the ecotype Col‐0 was used. All transgenic lines were generated from the Col‐0 as background. The transgenic lines for visualization of cell division and auxin distribution in the roots included *CyB1‐GFP*, *CYCD2,1‐GFP*, *pSHR::SHR‐GFP*, *pPLT1::PLT1‐YFP*, *pPLT2::PLT2‐YFP*, *DR5rev::GFP*, *R2D2*, *pPIN1::PIN1‐GFP*, *pPIN2::PIN2‐GFP*, *pPIN3::PIN3‐GFP*, *pPIN7::PIN7‐GFP*, *pAux1::Aux1‐YFP*, *DII::VENUS*, and *W131Y* used in this study were previously described;^[^ ^64^, ^94^, ^99^, ^100^, ^101^, ^102^, ^103^, ^104^ ^]^ the loss‐of‐function mutants *shr*, *plt1/plt2*, and *pin2* derived from Col‐0 background were also previously reported.^[^ ^105^, ^106^ ^]^

Plant transformations were performed as follows: the CDS of *PLT1*, *PLT2*, and *SHR* were cloned into the Gateway entry vector pDONR221 by BP reaction (Invitrogen). Primers used for amplification were as follows: PLT1‐CDS‐F (5′‐ATGAATTCTAACAACTGGCTTGGC‐3′) and PLT1‐CDS‐R (5′‐TTACTCATTCCACATAGTGAAAACACCAC‐3′) for *PLT1* (AT3G20840.1); PLT2‐CDS‐F (5′‐ ATGAATTCTAACAACTGGCTCGC‐3′) and PLT2‐CDS‐R (5′‐TTATTCATTCCACATCGTGAAAACACCT‐3′) for *PLT2* (AT1G51190.1); SHR‐CDS‐F (5′‐ATGGATACTCTCTTTAGACTAGTCAGT‐3′) and SHR‐CDS‐R (5′‐ TTACGTTGGCCGCCACGCA‐3′) for *SHR* (AT4G37650.1). Then the CDS of these genes were cloned to the binary vector pK7WG2 via LR reaction (Invitrogen). For plant transformation, the binary vectors carrying the CDS were introduced into *Agrobacterium tumefaciens* strain GV3101 (Weidibio, Shanghai, China) by electroporation. The floral dipping method were used for *Arabidopsis* transformation. Transgenic *Arabidopsis* plants were selected on half‐strength MS medium containing 50 mg mL^−1^ of Kanamycin. Homozygous T3 or T4 lines were subsequently used for experimental analysis.

### Plant Cultivation and Nanoplastics Exposure

For phenotypic analysis of *Arabidopsis* seedling, seeds (wild‐type and transgenic lines) were sterilized by soaking in 75% ethanol solution for 3 min, with occasional inversion. The seeds were then washed twice with 95% ethanol to remove residual water and transferred to a sterile filter paper to air‐dry. The surface sterilized seeds were sown on ^1^/_2_ MS medium and sealed with sterile tape. Seeds were vernalized at 4 °C for 48 h before being transferred to a growth chamber. Culture conditions were set at 22 °C, with a 16‐h light/8‐h dark cycle and a light intensity of ≈120 µmol m^−2^ s^−1^. Stock solutions of PS‐NPs were prepared in sterile water and stored at 4 °C as 1 mg mL^−1^ working solution. These were further diluted to the desired concentrations in the growth medium. Four‐day‐old seedlings were transferred to medium containing different sizes of PS‐NPs and allowed to grow for various periods for subsequent physiological or biochemical analysis.

### Observation of Root Tip's Structure and Biochemical Staining

Accumulation of PS‐NPs in plant roots was observed using Scanning Electron Microscope (Nano SEM‐450, FEI). Seedlings treated with PS‐NPs for 24 h were rinsed with deionized water, fixed in 2.5% glutaraldehyde at 4 °C for 24 h, and washed twice with 0.1 M pH 6.8 PBS buffer. The sample were dehydrated using an ethanol gradient of 30, 50, 70, 80, and 90% and anhydrous ethanol, followed by CO_2_ critical point drying. The samples were golden‐coated for 60 s (EMACE600, Leica) and examined under high vacuum at 5 kV.

To investigate root tissue structure, seedlings were stained with 5% propidium iodide (PI) for 5 min and root tip was excised for morphology examination. To observe starch granules in root tips, seedlings were stained with Lugol's solution for 2 min, washed twice with deionized water, mounted on coverslips with chloral hydrate solution (4 g chloral hydrate, 1 ml glycerol, 2 ml water) and observed immediately. The mPS‐PI staining method was used for staining starch in root tips, as previously reported.^[^ ^107^ ^]^ Seedlings were fixed in 50% methanol plus 10% acetic acid at 4 °C for 24 h. The root tissues were then washed with sterilized water and incubated in a solution with 1% periodate for 40 min, then the seeds were rinsed again with sterilized water and cultured in Schiff's reagent (0.1 m sodium metabisulphite, 0.15 N HCl) containing PI (100 µg ml^−1^) for 2 h until the plants had a visible staining. The plant samples were removed and put on slides, and the samples were covered with chloral hydrate solution and left overnight at room temperature conditions. Finally, the excess chloral hydrate solution was eliminated and Hoyer's solution (30 g acacia gum, 200 g chloral hydrate, 20 g glycerol, and 50 ml water) was applied as a sealer, covered with a coverslip, left for 3 days at least and then placed under a laser confocal microscope for observation.

Cell proliferation was assessed using EdU incorporation. In short, 4‐day‐old *Arabidopsis* seedlings were treated with PS‐NPs for 12 h, followed by transferred to ^1^/_2_ MS medium containing 1 µm EdU for additional 12 h. The seedlings were washed twice in ^1^/_2_ MS medium supplemented with 1% sucrose to remove excess EdU (2×5 min) and fixed twice in phosphate‐buffered saline (PBS, pH 7.4) containing 0.5% Triton X‐100 before EdU detection. EdU incorporation was detected using the Cell Proliferation Kit (Beyotime) and Alexa Fluor 488. Fluorescence was visualized using confocal microscopy.

### Confocal Microscopy Imaging

Confocal imaging was performed with a Leica Stellaris 8 laser confocal microscope using a 440‐790 nm white light laser. Seedlings were stained with 5% propidium iodide (PI) for 5 min and briefly washed with double‐distilled water. Fluorescent signals were imaged using different excitation/emission parameters (*E_x_*/*E_m_*): GFP 488/507, PI 535/615, YFP 513/527, RFP 558/608, VENUS 515/528, and Alexa Fluor 488: 488/519 nm. The mean intensity of fluorescence signal was quantified using stack profile of LAS X software.

### Transcriptomics Analysis

Roots of 4‐day‐old wild‐type seedlings exposed to PS‐NPs (40 or 100 µg ml^−1^ of 20, 100, and 500 nm sizes) were collected, washed three times with deionized water, and quickly frozen in liquid nitrogen for RNA extraction. Total RNA was isolated using TRIzol reagent, with three biological replicates of at least 200 seedlings per replicate. RNA sequencing was performed using the Illumina NovaSeq 6000 platform (Shanghai OE Biotech Co., Ltd.), and differentially expressed genes (DEGs) analysis was carried out as previously described. Detailed methods were provided in the Texts S3‐S4 (Supporting Information).

### Fluorescence Recovery After Photobleaching (FRAP)

For fluorescence recovery after photobleaching (FRAP), transgenic *pPIN2::PIN2‐GFP* seedlings were grown on ^1^/_2_ MS medium with PS‐NPs for 24 h. The diffusion rate of PIN2‐GFP within the plasma membrane was assessed by bleaching specific regions of interest (ROIs) in the root epidermal cells using a Leica Stellaris 8 confocal microscope (https://www.leica‐microsystems.com/science‐lab/life‐science/step‐by‐step‐guide‐for‐frap‐experiments/). Fluorescence recovery was monitored for 7 min after bleaching, with images captured every minute. The average fluorescence intensity recovery of the ROI was measured using Leica software, with at least three independent roots analyzed per condition.

### Gravitropic Growth Analysis

To test gravitropic response, 4‐day‐old wild‐type seedlings were placed on ^1^/_2_ MS medium with PS‐NPs and oriented vertically for 24 h. After rotating the petri dishes by 90°, seedlings were allowed to grow vertically for several hours, with images captured at 3, 6, and 18 h. Root bending curvatures were analyzed using Image J.

For auxin redistribution analysis in response to gravistimulation, 4‐day‐old seedlings of *Arabidopsis* transgenic line *DII::VENUS* and *DR5rev::GFP* were treated with PS‐NPs and subjected to gravistimulation. After rotating by 90°, the fluorescence intensity of VENUS and GFP was separately visualized using a Leica Stellaris 8 confocal microscope at different time intervals to determine auxin redistribution.

### Statistical Analysis

Primary root elongation and root curvature angle were analyzed using Image J, and fluorescence intensity of GFP and VENUS was determined by LAS X. Data were processed using GraphPad Prism 9, and statistical significance was determined using one‐way ANOVA by Dunnett's multiple comparisons post hoc test. Data were presented as mean ± standard deviation, with significance defined as ∗∗∗, *p* < 0.001; ∗∗, *p* < 0.01; ∗, *p* < 0.05; ns, not significant, *p* > 0.05.

## Conflict of Interest

The authors declare no conflict of interest.

## Author Contributions

S.M. and Z.H. contributed equally to this work. X.L., Y.Z., and X.G. designed the supervised the project. S.M. and Z.H. performed most of the experiments. S.M. wrote the manuscript with input from all the authors. C.T., X.Q., and L.D. conducted the experiments. X.L., Y.Z., and X.G. revised the manuscript.

## Supporting information



Supporting Information

## Data Availability

The data that support the findings of this study are available from the corresponding author upon reasonable request.
