# Supplementary material for: Root Meristem Maintenance Mechanisms are Key to Plant Defense Against Nanoplastics
Source: Adv Sci (Weinh). 2025 Aug 26;12(43):e11837. doi: 10.1002/advs.202511837 (PMC12631816; doi:10.1002/advs.202511837)
Supplement: Supplementary file 1 — Supporting Information [file ADVS-12-e11837-s001.docx]

Supporting Information

**Root meristem maintenance mechanisms are key to plant defense against nanoplastics**

*Sirui Ma^a, #^, Zhengdong Hua^a, #^,* *Chenglong Tang^b^, Xinran Qiu^a^, Ling Ding^a^, Xujun Liang^a^, Yuzhou Zhang^b *^ , Xuetao Guo^a *^*

^a^ College of Natural Resources and Environment, Northwest A&F University, Yangling, Shaanxi, 712100, China

^b^ State Key Laboratory of Crop Stress Resistance and High - Efficiency Production, College of Life Sciences, Northwest A&F University, Yangling, Shaanxi, 712100, China

^#^These authors contributed equally: Sirui Ma and Zhengdong Hua

*Corresponding authors.

E-mail addresses: guoxuetao2005@nwafu.edu.cn (X. Guo).

yuzhou.zhang@nwafu.edu.cn (Y. Zhang)

**Text S1 Scanning electron microscopy and Transmission electron microscopy observation method of PS-NPs**

In this study, the particle size and morphology of PS-NPs were characterized by field emission scanning electron microscopy (Nova Nano SEM-450, FEI, Czech Republic) and transmission electron microscope (Hitachi HT7800, Japan), respectively. The procedure of SEM test was as follows: a small amount of PS-NPs suspension was taken and dropped on a copper sheet adhered with conductive adhesive, and then vacuum sprayed with gold for 2 min after drying naturally, with an operating voltage of 5 kV. TEM was also used to observe the size of the nanoplastics as follows: a small amount of plastic suspension (50 mg/L) was dropped onto a 200-mesh carbon-supported copper mesh using a 200-microliter pipette gun, and left to dry spontaneously at room temperature and protected from light and then the morphology was observed at an operating voltage of 80 kV. The images were taken at different resolutions.

**Text S2 Determination of hydrodynamic diameter**

The hydrodynamic particle size distribution of PS-NPs suspensions with different particle sizes was determined using a Malvern nanoparticle sizer (ZEN3600, UK). To maintain the dispersion, the suspensions were subjected to ultrasonication at a power of 100 w and a frequency of 40 Hz for 15 min. Afterwards, it was measured after standing and cooling at room temperature. The specific parameters were as follows: equilibration time 120 s, measurement temperature 25 ℃.

**Text S3 mRNA sequencing experimental method**

Total RNA was extracted using the TRIzol reagent (Invitrogen, CA, USA) according to the manufacturer’s protocol. RNA purity and quantification were evaluated using the NanoDrop 2000 spectrophotometer (Thermo Scientific, USA). RNA integrity was assessed using the Agilent 2100 Bioanalyzer (Agilent Technologies, Santa Clara, CA, USA). Then the libraries were constructed using VAHTS Universal V6 RNA-seq Library Prep Kit according to the manufacturer’s instructions. The transcriptome sequencing and analysis were conducted by OE Biotech Co., Ltd. (Shanghai, China).

**Text S4 mRNA sequencing analysis process**

The libraries were sequenced on a llumina Novaseq 6000 platform and 150 bp paired-end reads were generated. About 50 M raw reads for each sample were generated. Raw reads of fastq format were firstly processed using fastp and the low-quality reads were removed to obtain the clean reads. Then about 48 M clean reads for each sample were retained for subsequent analyses. The clean reads were mapped to the *Arabidopsis thaliana* genome using HISAT2 ^[1, 2]^. FPKM of each gene was calculated and the read counts of each gene were obtained by HTSeq-count4. PCA analysis were performed using R (v 3.2.0) to evaluate the biological duplication of samples ^[3, 4]^.

Differential expression analysis was performed using the DESeq2 ^[5]^. Q value < 0.05 and foldchange > 1.5 or foldchange < 0.5 was set as the threshold for significantly differential expression gene (DEGs). Hierarchical cluster analysis of DEGs was performed using R (v 3.2.0) to demonstrate the expression pattern of genes in different groups and samples. The radar map of top 30 genes was drawn to show the expression of up-regulated or down-regulated DEGs using R packet ggradar.

Based on the hypergeometric distribution, Gene Ontology (GO) and Kyoto Encyclopedia of Genes and Genomes (KEGG) pathway enrichment analysis of DEGs were performed to screen the significant enriched term using R (v 3.2.0), respectively. R (v 3.2.0) was used to draw the column diagram and bubble diagram of the significant enrichment term ^[6]^.

**Table S1 The table of ^1^/_2_ MS medium component**

| Reagent name | Composition (mg/L) |
| --- | --- |
| KNO_3_ | 950 |
| KH_2_PO_4_ | 85 |
| NH_4_NO_3_ | 825 |
| MgSO_4_ | 185 |
| CaCl_2_ | 220 |
| H_3_BO_3_ | 6.2 |
| KI | 0.83 |
| MnSO_4_ | 22.3 |
| ZnSO_4_ | 8.6 |
| Na_2_MoO_4_ | 0.25 |
| CoCl_2_ | 0.025 |
| CuSO_4_ | 0.025 |
| FeSO_4_ | 27.8 |
| Inositol | 100 |
| Glycine | 2 |
| Thiamine hydrochloride | 0.1 |
| nicotinic acid | 0.5 |
| pyridoxine hydrochloride | 0.5 |
| EDTA disodium salt | 37.3 |

**Table S2 The hydrodynamic diameter of different PS-NPs**

| PS-NPs | Size (nm) |
| --- | --- |
| 20 | 23.31±0.28 |
| 100 | 98.46±0.33 |
| 200 | 237.97±0.90 |
| 500 | 532.73±19.78 |
| 1000 | 1302.67±12.58 |

**
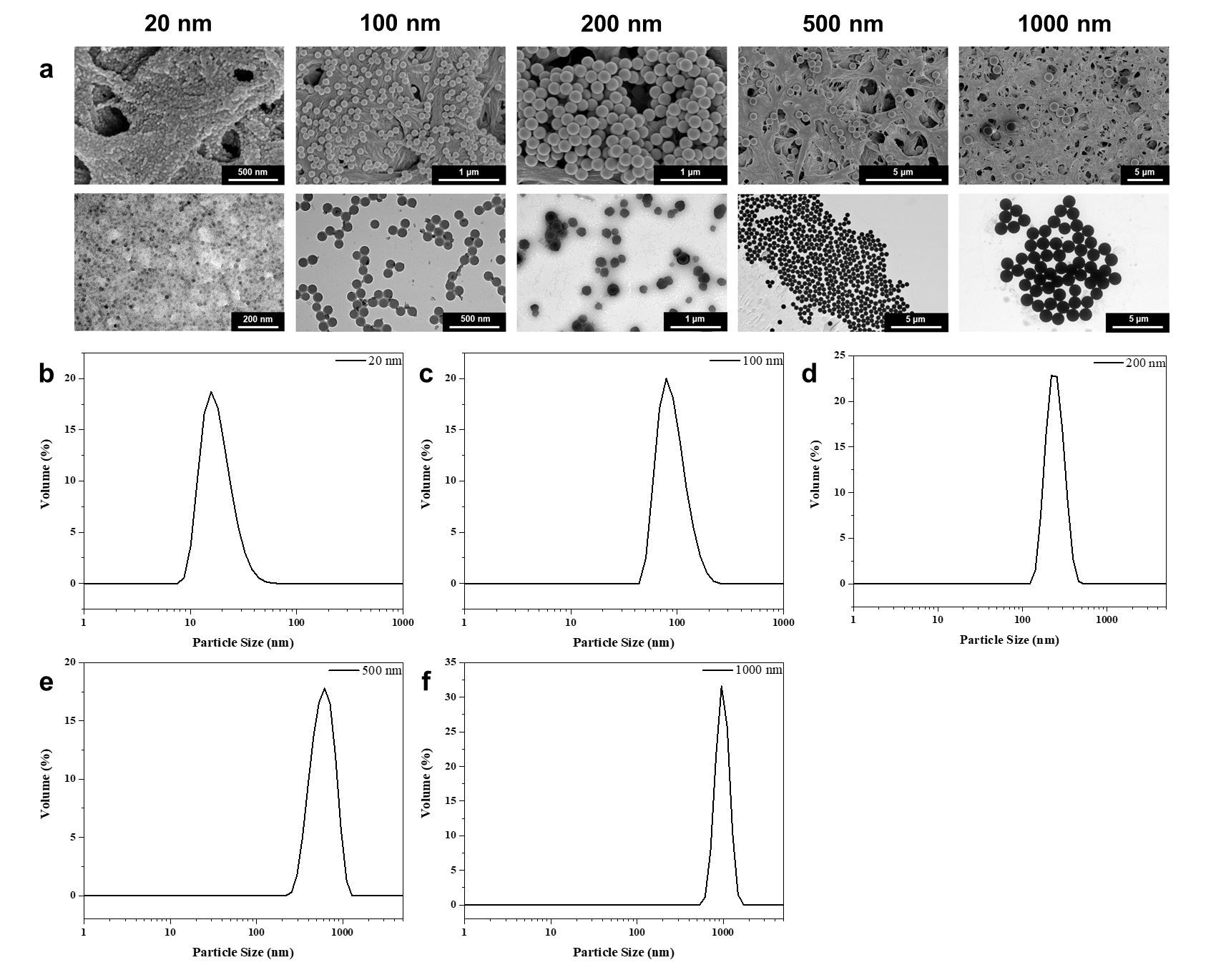
**

**Figure S1. Characterization of nanoplastics with different sizes.**

a, The images of SEM in the upper panel and TEM in the lower panel images jointly show the nanoplastics with different sizes.

b, The hydrodynamic diameter determined by dynamic light scattering (DLS) for nanoplastics.


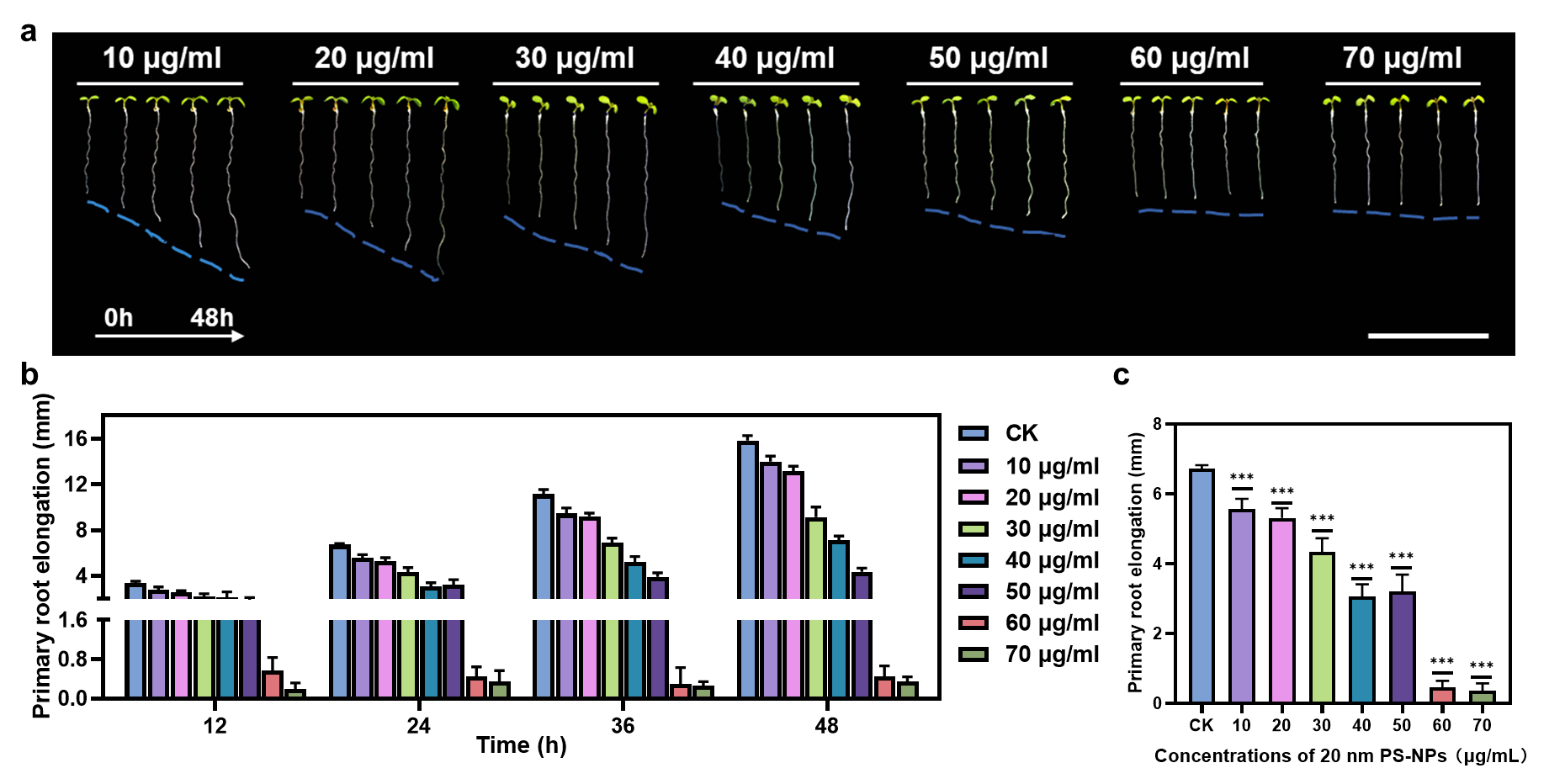


**Figure S2. The effect on primary root growth under various concentrations of 20 nm PS-NPs treatments for 48 h.**

a, Phenotypic images of primary root growth of *Arabidopsis* seedlings exposed to different concentrations of 20 nm PS-NPs. The white arrow indicated that images were acquired every 12 h. Scale bar 2 cm.

b, Quantitative analysis of primary root elongation at different time points under 20 nm PS-NPs treatment.

c, Changes in primary root elongation after 24 h of treatment with different concentrations of 20 nm PS-NPs

Data were expressed as means ± SD. Statistical analysis was performed through a one-way ANOVA followed by Dunnett's multiple comparisons test. **p* <0.05, ***p* <0.01, ****p* <0.001.


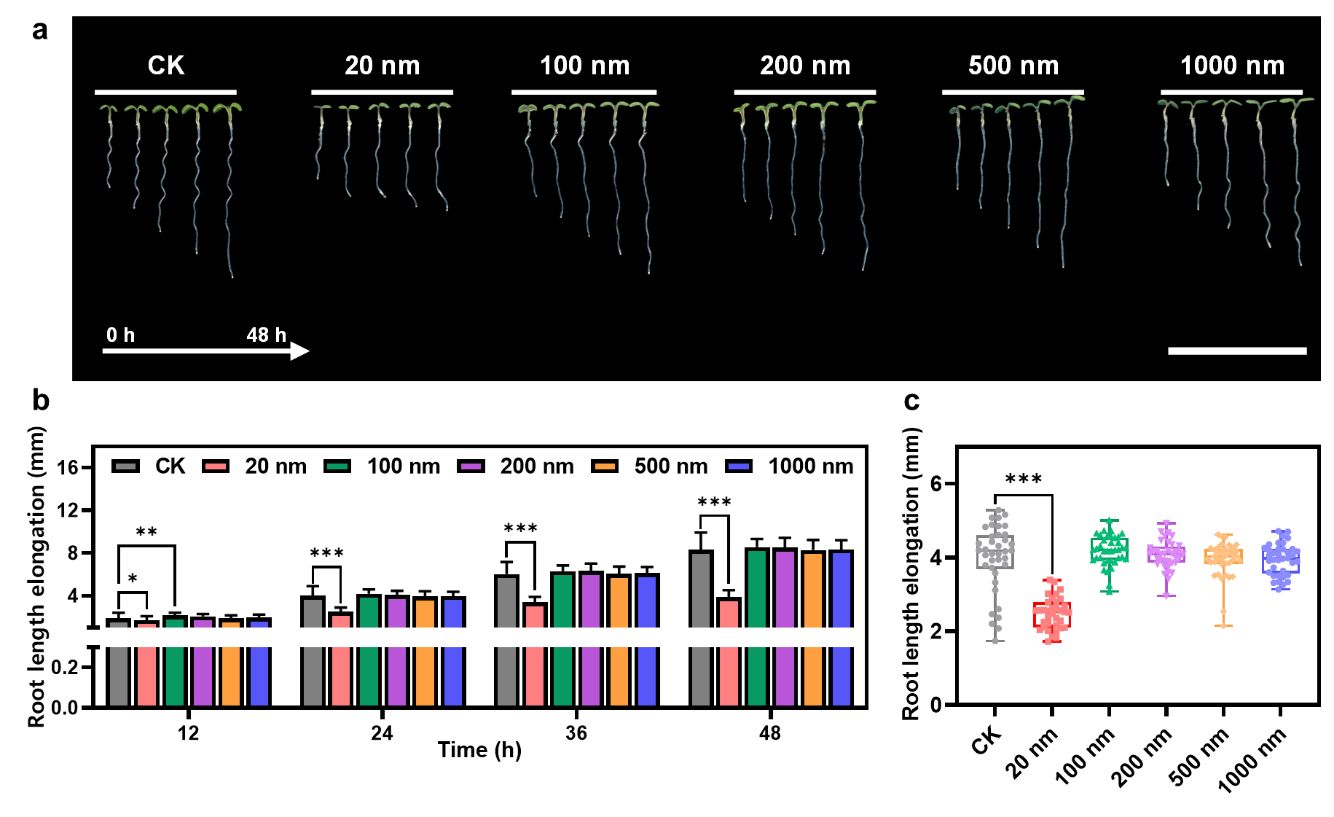


**Figure S3. The effect of exposure to PS-NPs with different sizes of 40 μg/ml on *Arabidopsis thaliana* growth within 48h.**

The phenotype of treated seedlings (a), and histogram of root length elongation at different time intervals (b) and box plot at 24h (c). The white arrow indicated that images were acquired every 12 h. Scale bar 1.5 cm. Data were expressed as means ± SD. Statistical analysis was performed through a one-way ANOVA followed by Dunnett's multiple comparisons test. **p* <0.05, ***p* <0.01, ****p* <0.001.


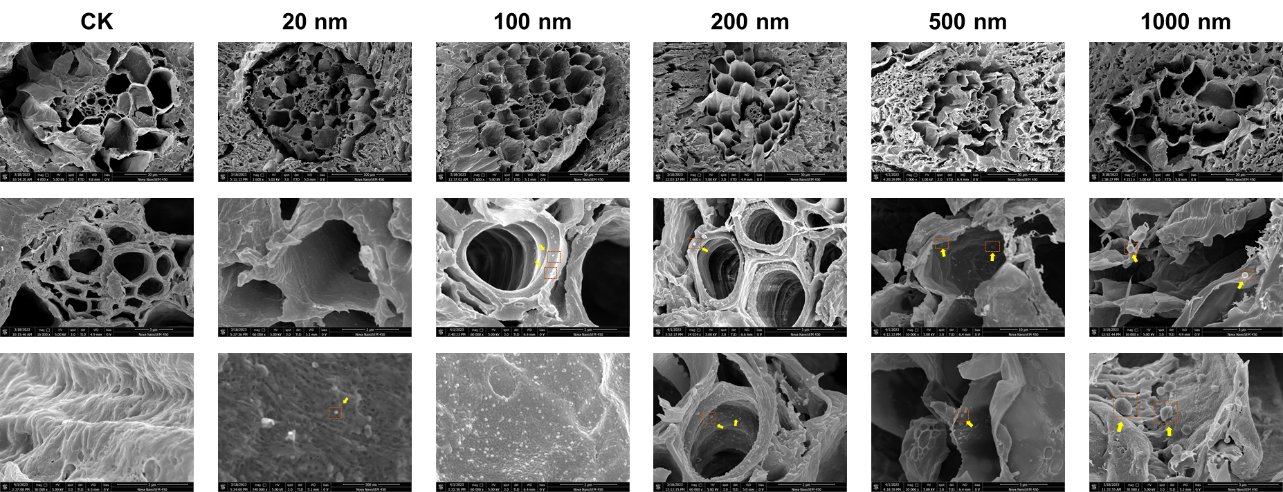


**Figure S4. SEM images of different PS-NPs in the root.**

SEM images of cross sections of 4-days-old Col-0 seedlings roots exposed to 40 μg/ml PS-NPs with different size treatments for 48 h.


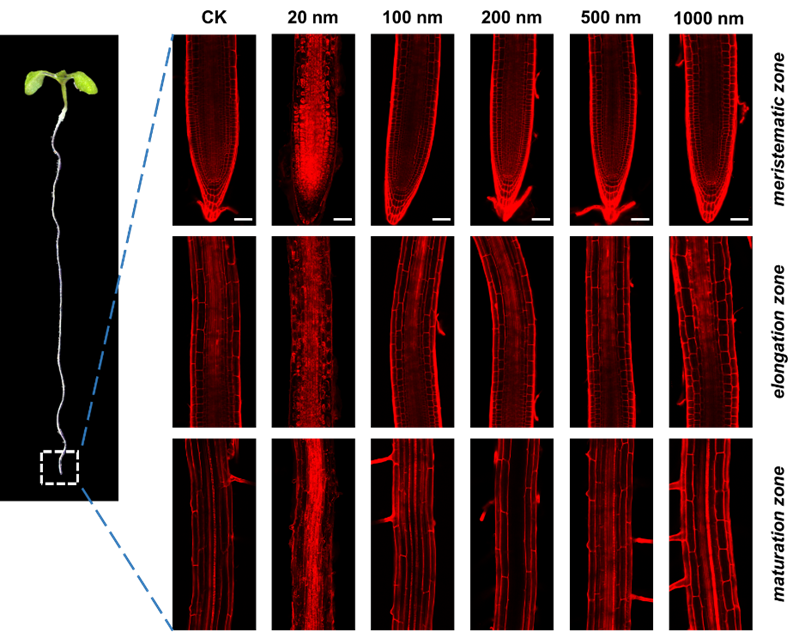


**b**

**a**


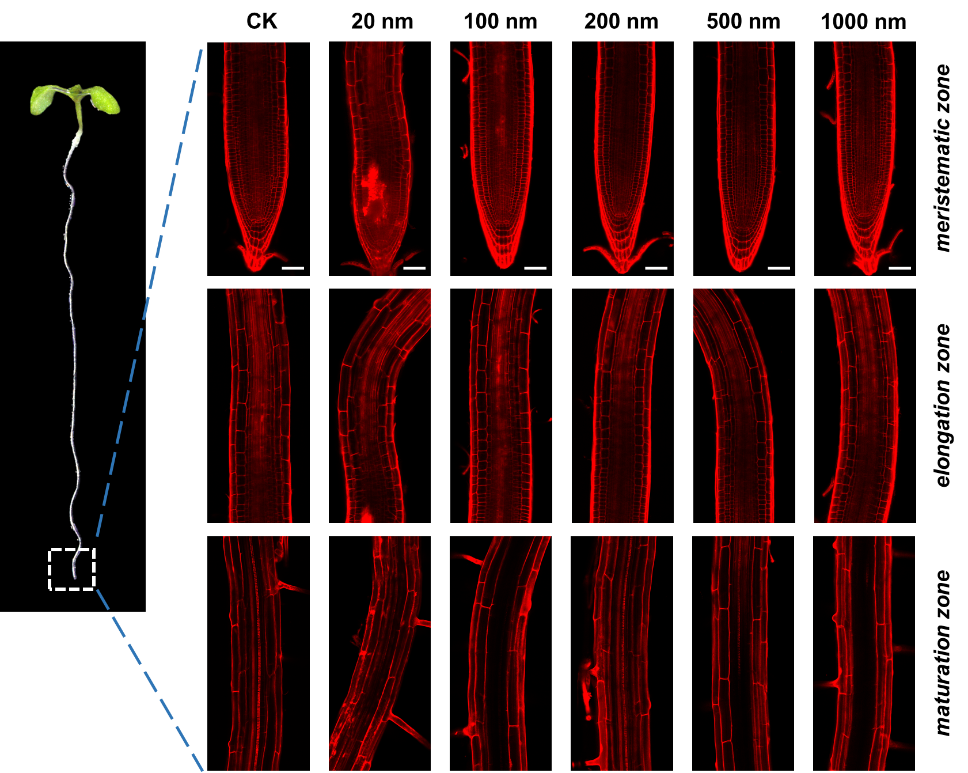


**Figure S5. Determining primary root development after exposure to different PS-NPs using PI staining.**

PI staining images of the apical tissues exposed to 100 (a) or 40 (b) μg/ml PS-NPs with size of 20, 100, 200, 500 and 100 nm for 48h. Scale bar 50 μm.


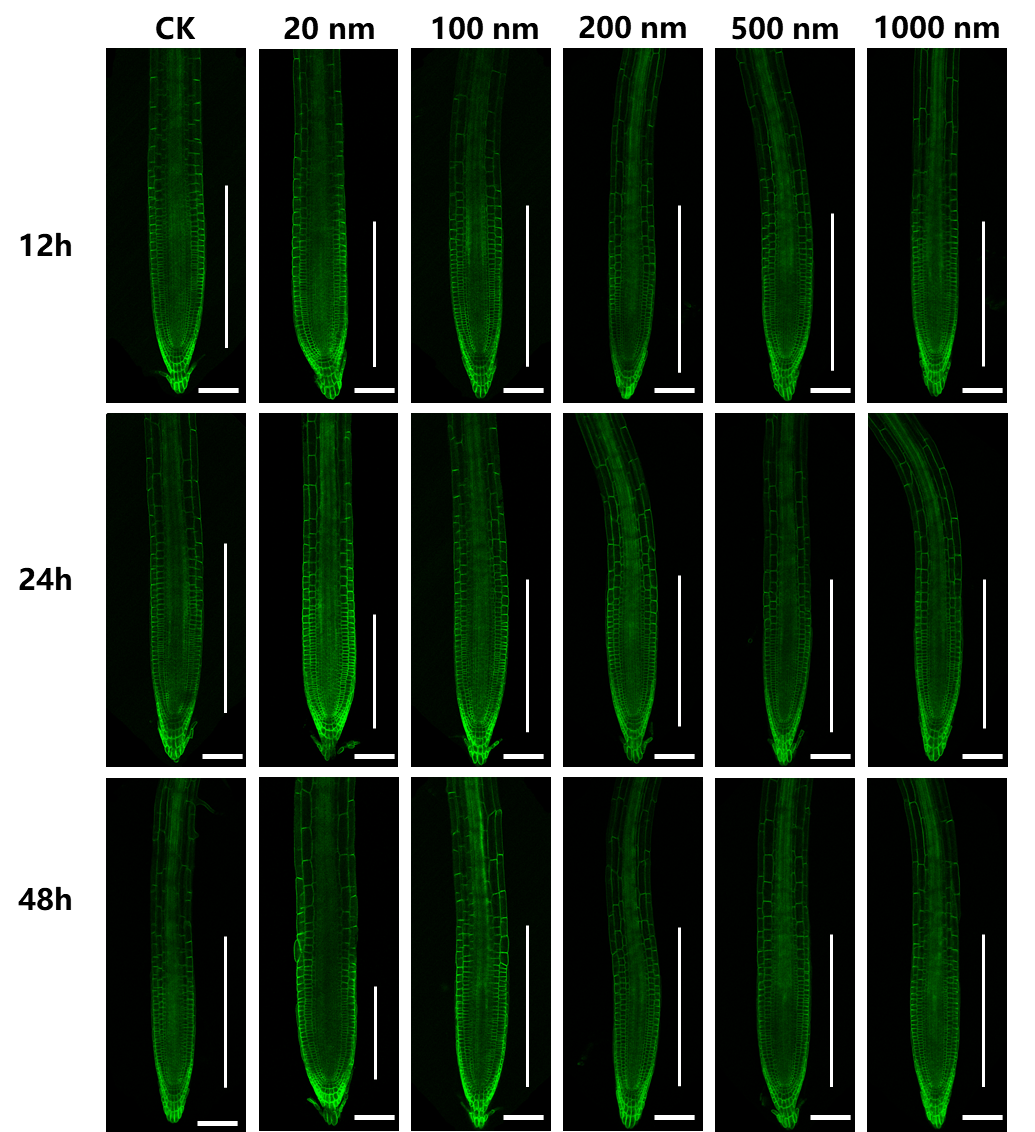


**Figure S6.** **Confocal fluorescence images of *W131Y* treated with different PS-NPs.**

Response of transgenic line *W131Y* in the root tips of 4-day-old seedlings grown in the presence of 40 μg/ml PS-NPs with size of 20, 100, 200, 500 and 1000 nm for 48h. Scale bar 100 μm.


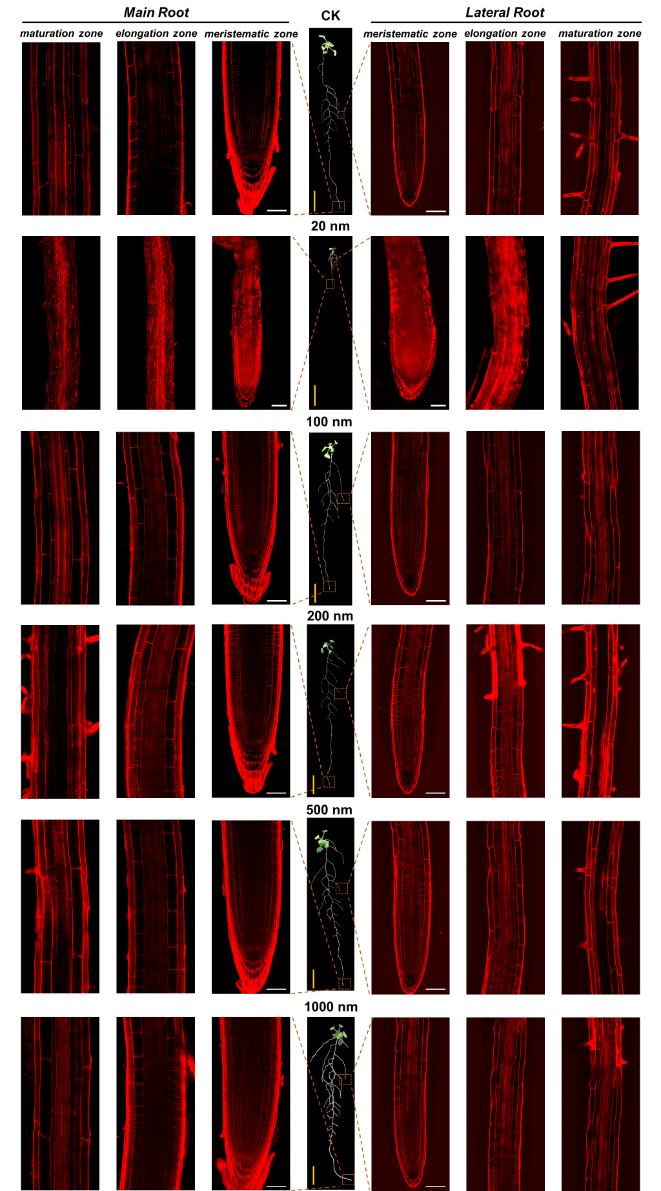


**Figure S7.** **Observation of PI staining for the development of primary and lateral roots of plants after prolonged exposure to different PS-NPs.**

Representative PI staining images of the apical tissues of primary and lateral roots of 4-days-old wild type seedlings after 8 days incubation exposed to PS-NPs with size of 20, 100, 200, 500 and 100 nm for 48h. The white and yellow scale bar were 50 μm and 1 cm, respectively.


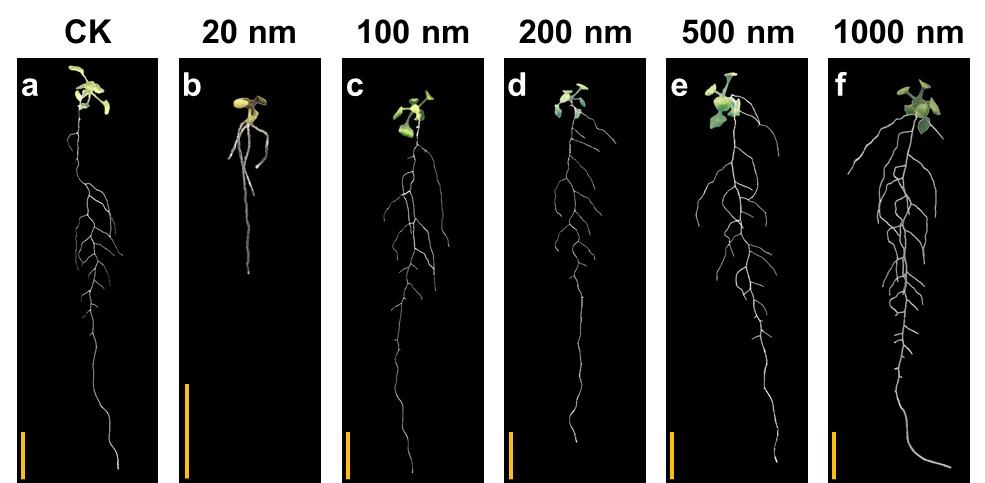


**Figure S8. Phenotypic images of plant roots systems following chronic exposure to different PS-NPs.**

Phytotoxicity of *Arabidopsis thaliana* upon exposure grown in ^1^/_2_ MS medium containing different size PS-NPs with concentration of 100 μg/ml for 8 d, respectively. Scale bar 1 cm.


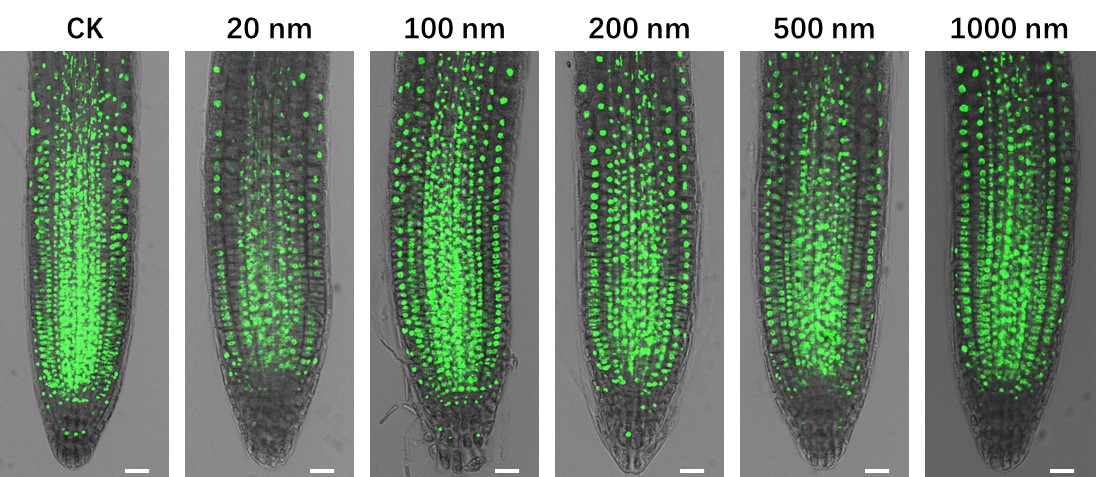


**Figure S9. EdU staining of root tips indicates cell division activity under different PS-NPs treatments.**

The EdU staining of root tips in Col-0 treated with control or 40 μg/ml of different particle sizes of PS-NPs for 24 h. Scale bar 25 μm.


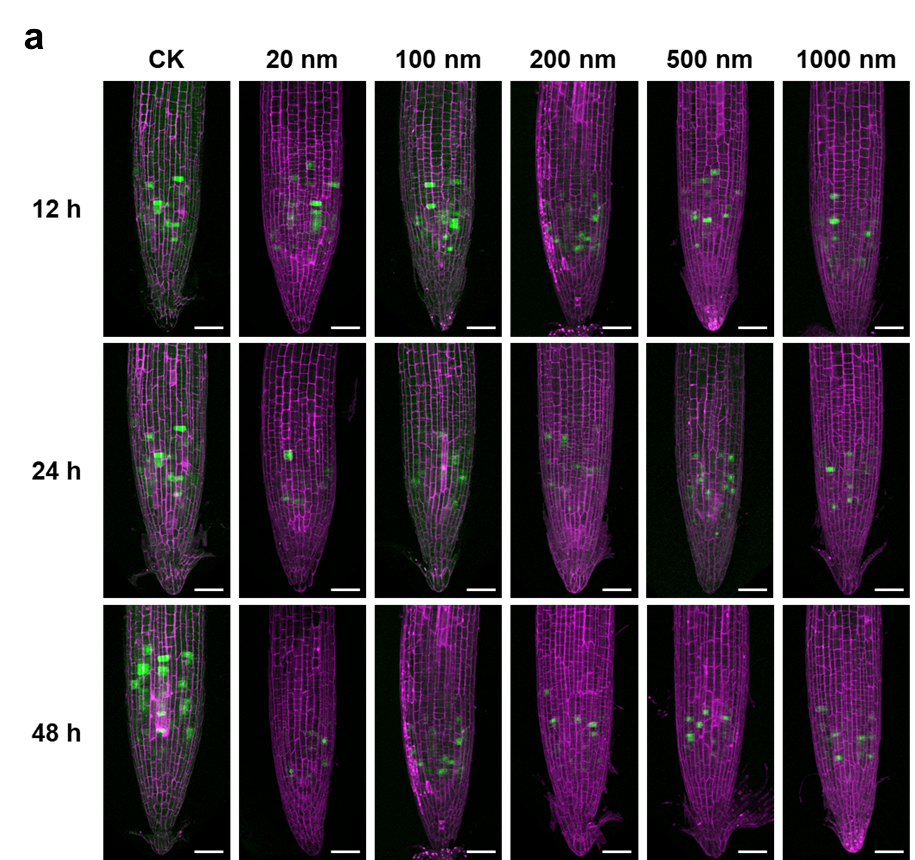


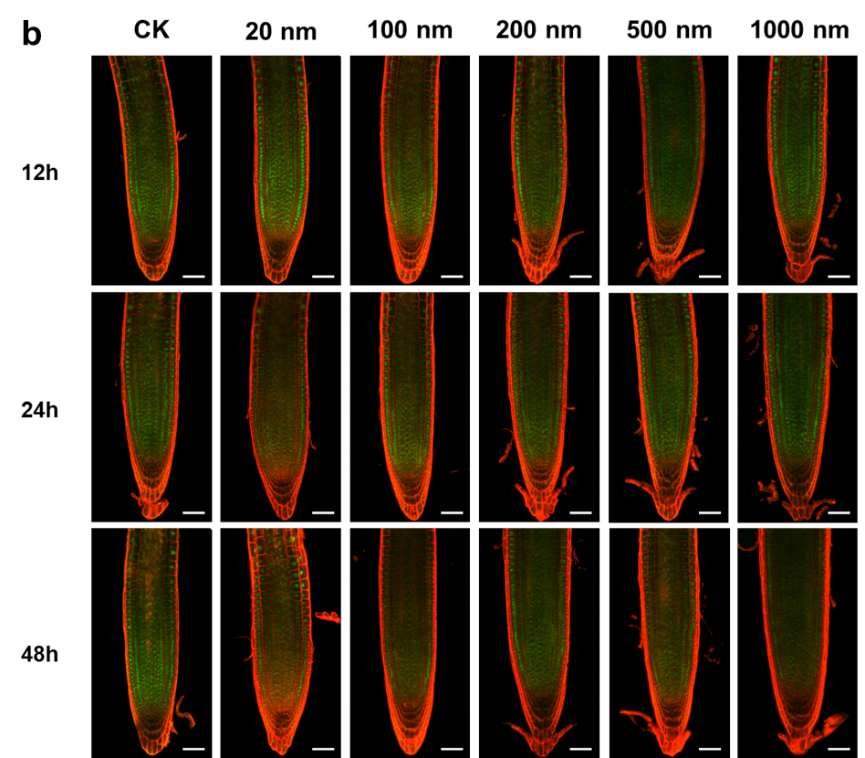


**Figure S10. Confocal fluorescence images of cell division markers treated with different PS-NPs in plant roots.**

The expression of fluorescent protein in roots of 4-day-old seedlings of transgenic lines *CyB1-GFP* (a) and *CYCD2,1-GFP* (b) grown for 48 h in medium containing 40 μg/ml of different particle sizes PS-NPs. Scale bar 50 μm.


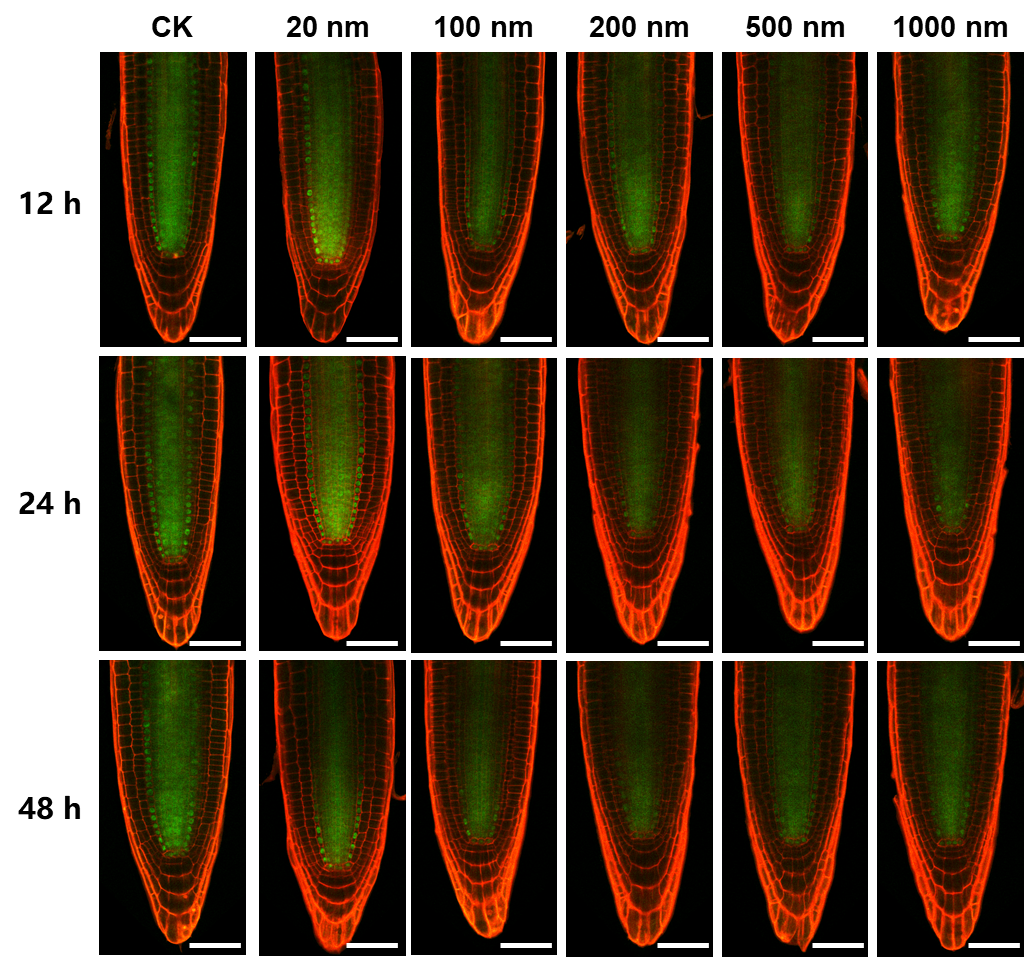


**Figure S11.** **Confocal fluorescence images of *pSHR::SHR-GFP* treated with different PS-NPs in plant roots.**

Influence of exposure to 40 μg/ml of PS-NPs with different particle sizes on expression levels of the transgenic line *pSHR::SHR-GFP* after incubation for 48 hours. Scale bar 50 μm.


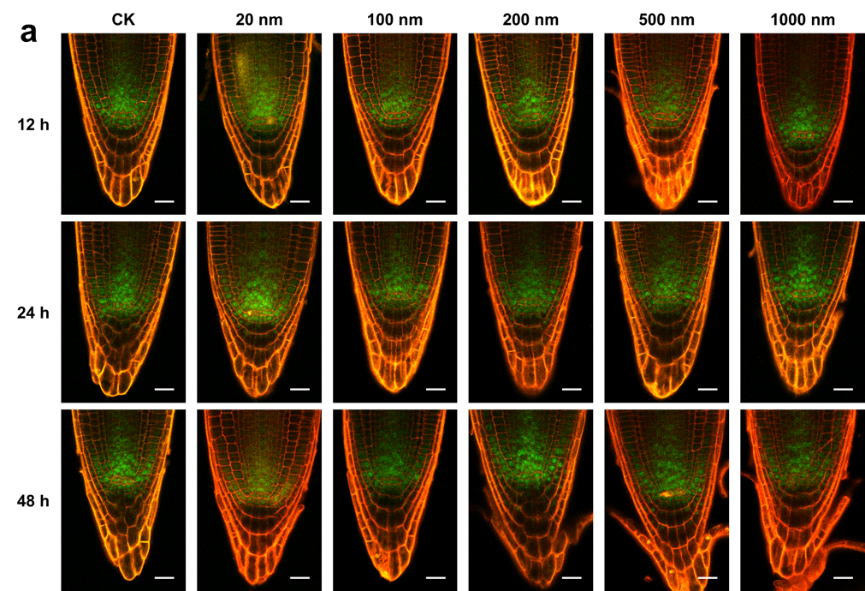


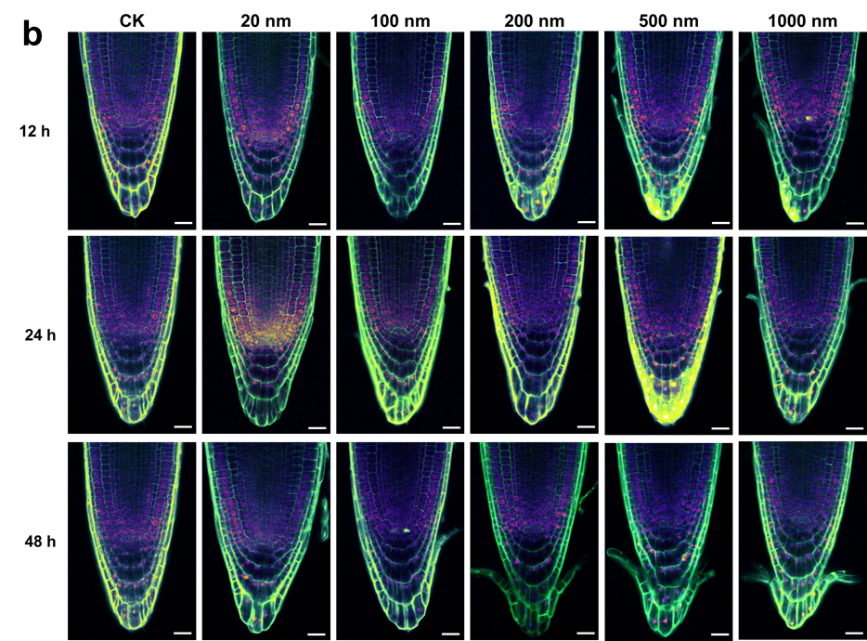


**Figure S12.** **Confocal fluorescence images of *pPLT1::PLT1-YFP* and *pPLT2::PLT2-YFP* treated with different PS-NPs in plant roots.**

The expression of transgenic lines *pPLT1::PLT1-YFP* (a) as well as *pPLT2::PLT2-YFP* (b) in the root tip meristematic tissue zone during growth treated with different particle sizes of PS-NPs at 40 μg/ml for 48h. Scale bar 20 μm.


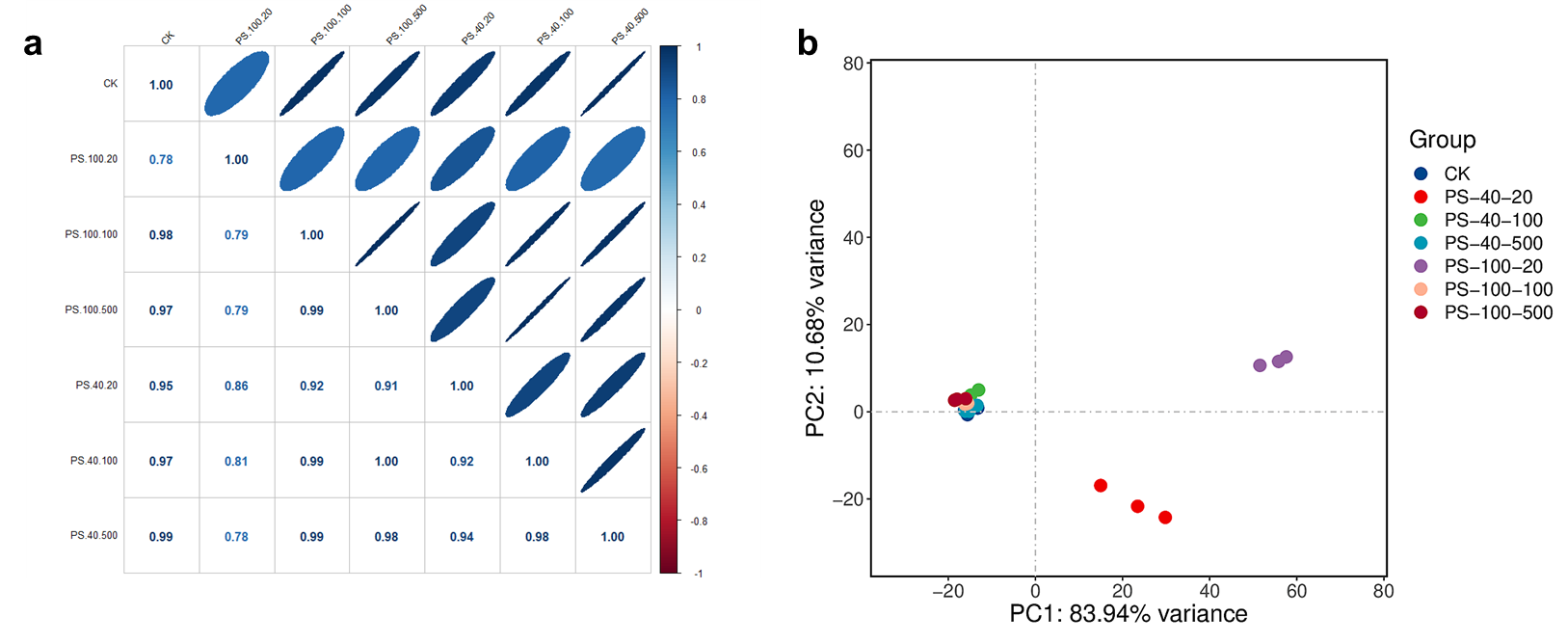


**Figure S13. Exposure to 20 nm PS-NPs alters gene expression patterns in plant roots.**

a, Correlation analysis of samples for transcriptomics.

b, Principal component analysis (PCA) was used to compare the different groups.


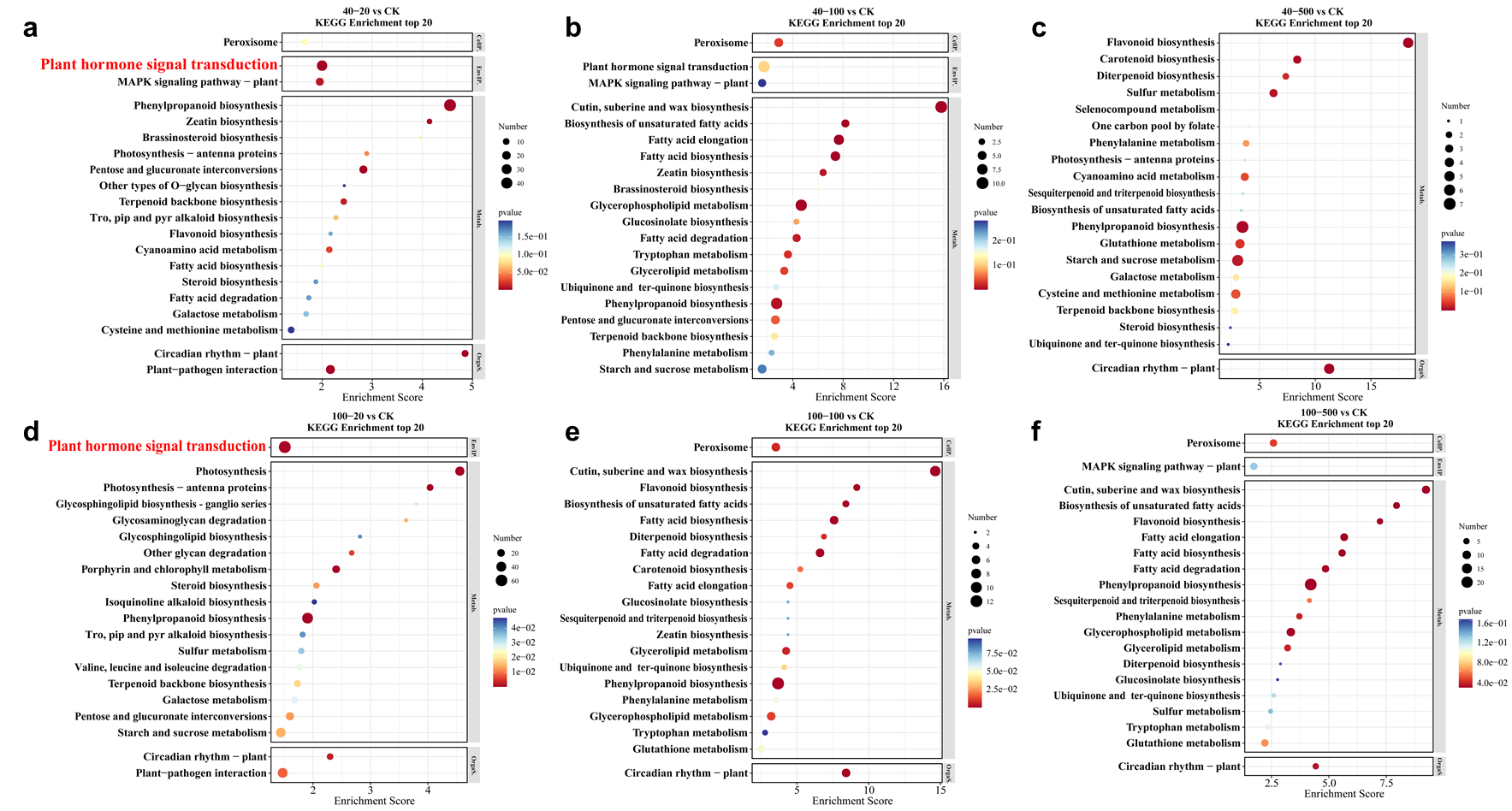


**Figure S14. KEGG pathway enrichment analysis of DEGs treated with different PS-NPs.**

The top 20 significantly enriched KEGG pathways in *Arabidopsis* roots in each treatment group under 40 or 100 μg/ml PS-NPs treatment. 20、100、500 nm PS-NPs for 40 μg/ml (a-c) or for 100 μg/ml (d-f). The size and color of the dots represent the gene number and the range of the -log_10_ ^(pvalue)^, respectively.


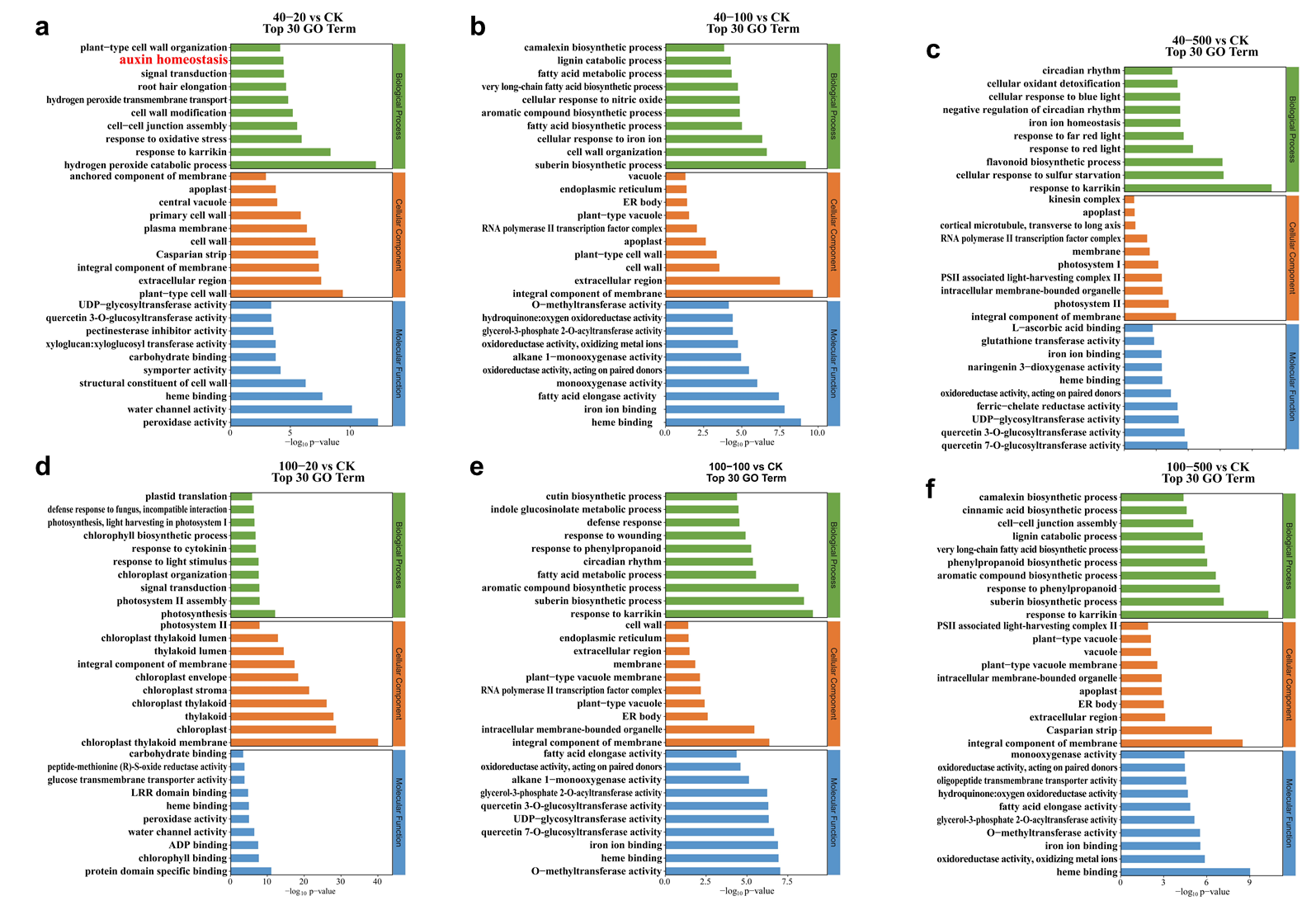


**Figure S15. GO pathway enrichment analysis of DEGs treated with different PS-NPs.**

The top 30 significantly enriched Gene ontology categories to DEGs in response to various treatments. 20、100、500 nm PS-NPs vs CK for 40 μg/ml (a-c) or for 100 μg/ml (d-f). The results are summarized in three GO categories: biological process, cellular component, and molecular function.


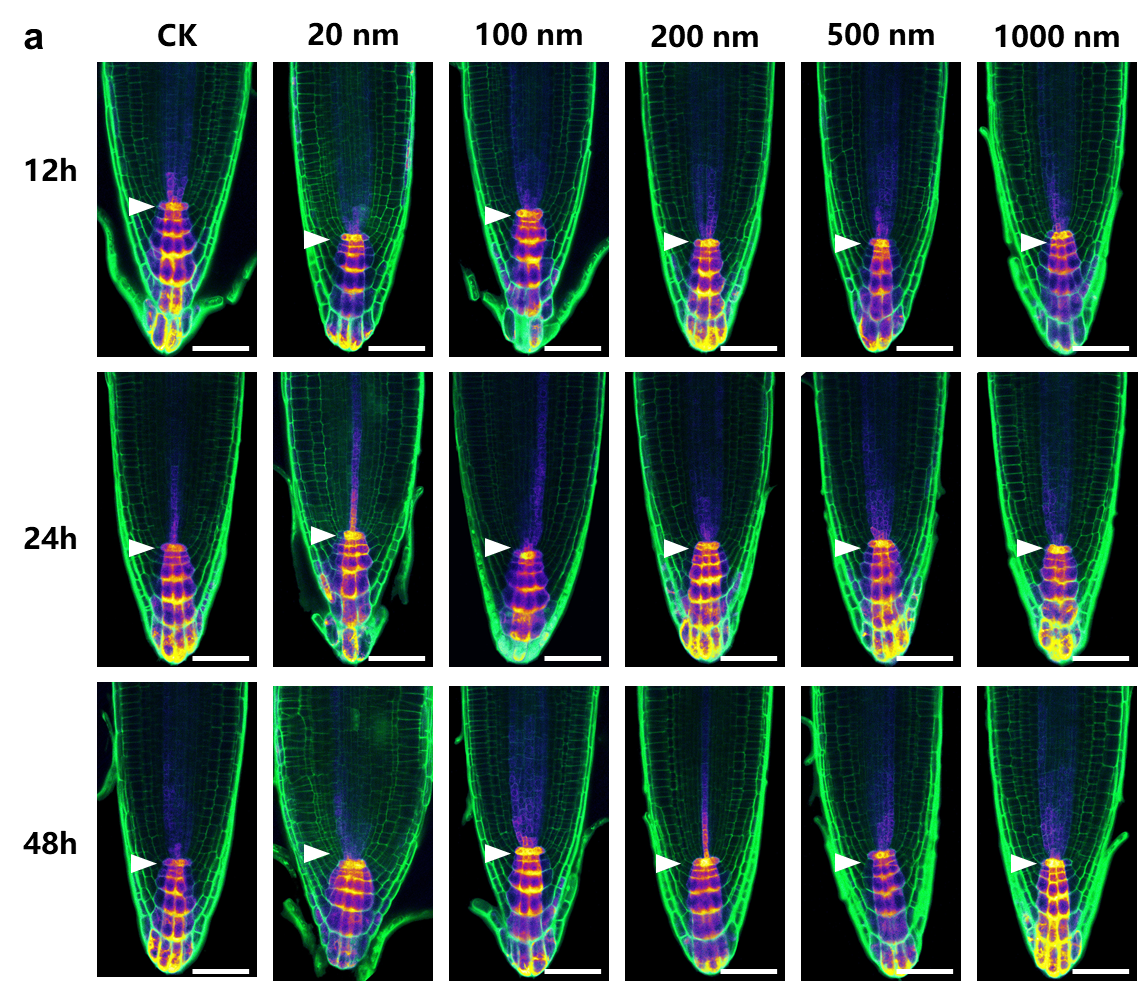


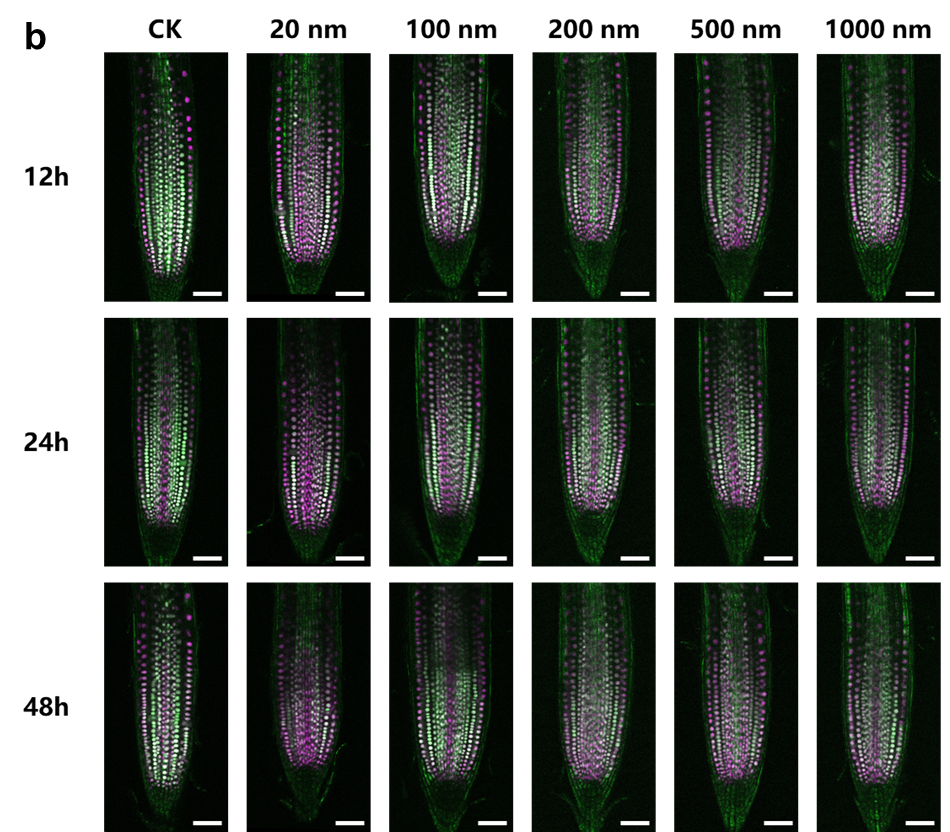


**Figure S16. Confocal fluorescence images of auxin responsive reporters *DR5rev::GFP* or *R2D2* treated with different PS-NPs in plant roots.**

The expression of transgenic lines *DR5rev::GFP* (a) as well as *R2D2*(b) in the root tip meristematic tissue zone treated with different particle sizes of PS-NPs at 40 μg/ml for 48h. Scale bar 50 μm.


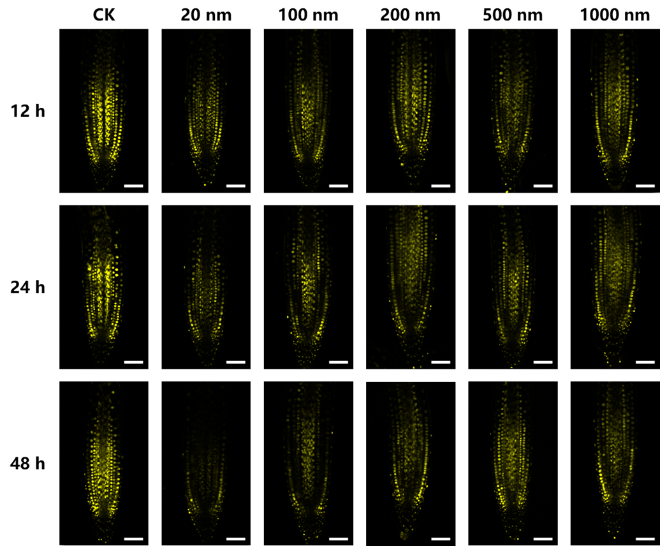


**Figure S17. Confocal fluorescence images of *DⅡ::VENUS* treated with different PS-NPs in plant roots.**

The expression of fluorescent protein in roots of 4-day-old seedlings of transgenic lines *DⅡ::VENUS* grown for 48 h in medium containing 40 μg/ml of different particle sizes PS-NPs. Scale bar 50 μm.


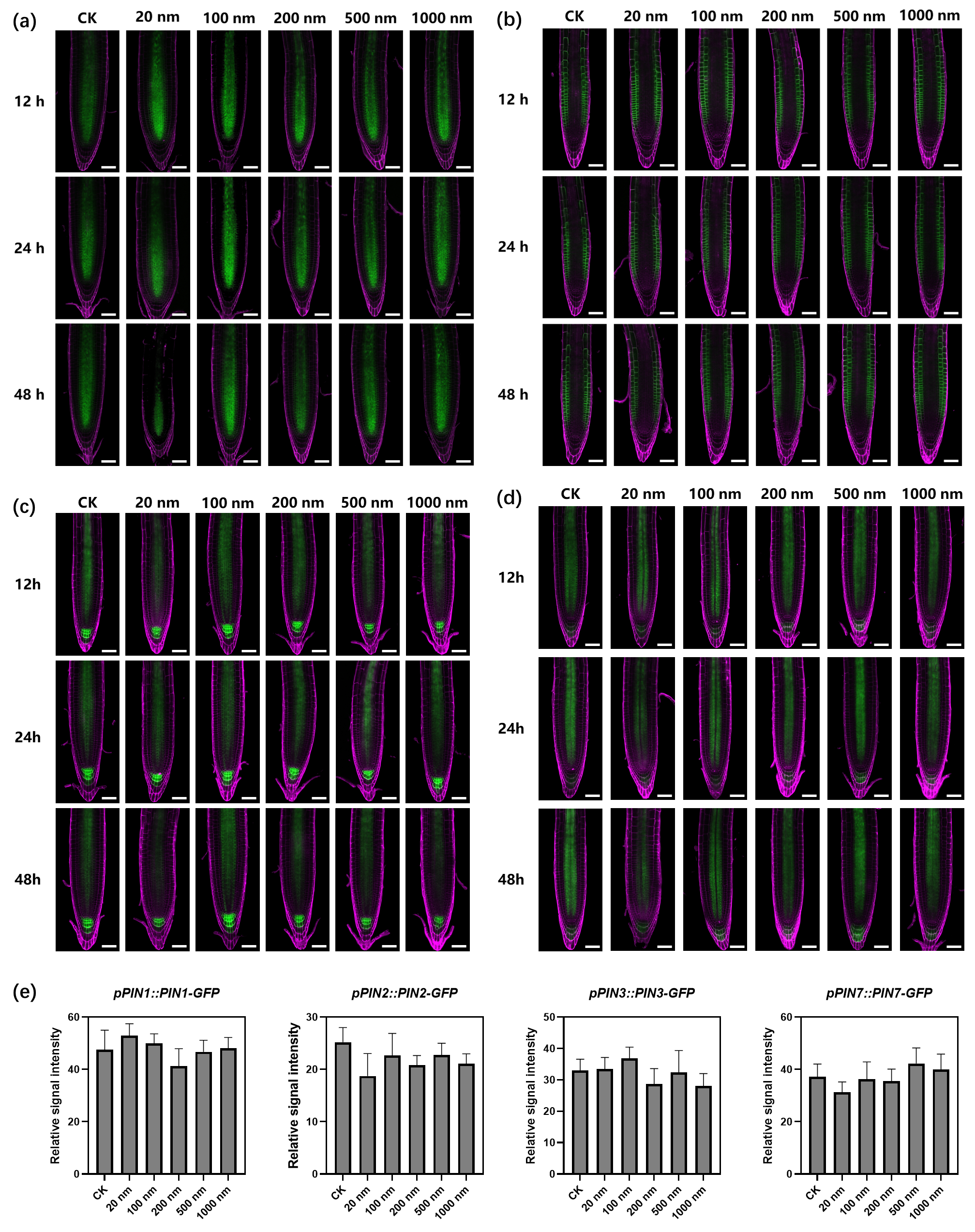


**Figure S18. Confocal fluorescence images of PIN proteins (auxin efflux carriers) treated with different PS-NPs in plant roots.**

The expression of green fluorescent protein in root tips at different time intervals during 48 h of growth of 4-day-old *pPIN1::PIN1-GFP* (a), *pPIN2::PIN2-GFP* (b), *pPIN3::PIN3-GFP* (c), and *pPIN7::PIN7-GFP* (d) transgenic seedlings in ^1^/_2_ MS medium containing 40 μg/mL of PS-NPs with different particle sizes. e, Quantification of signal intensity in auxin transporter (PIN1, PIN2, PIN3, PIN7) after 24 h of exposure to nanoplastics. Scale bar 50 μm.


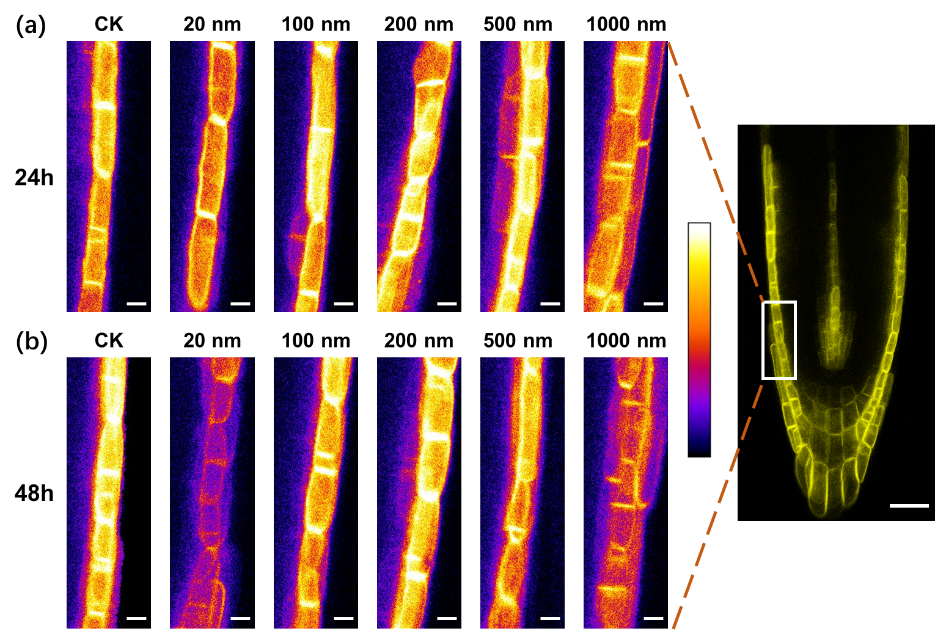


**Figure S19. Expression of auxin influx carrier AUX1/LIKE-AUX1 (AUX/LAX) proteins treated with different PS-NPs in plant roots.**

Confocal images of fluorescent protein expression in root epidermal cells of 4-day-old *pAux1::Aux1-YFP* seedlings grown at 40 μg/ml with different particle sizes of PS-NPs exposed for 24 h (a) and 48 h (b), respectively. The YFP channel images are shown in pseudo color, and the intensity scale is shown at the right. (White, high; Black, low). Scale bar 5 μm.


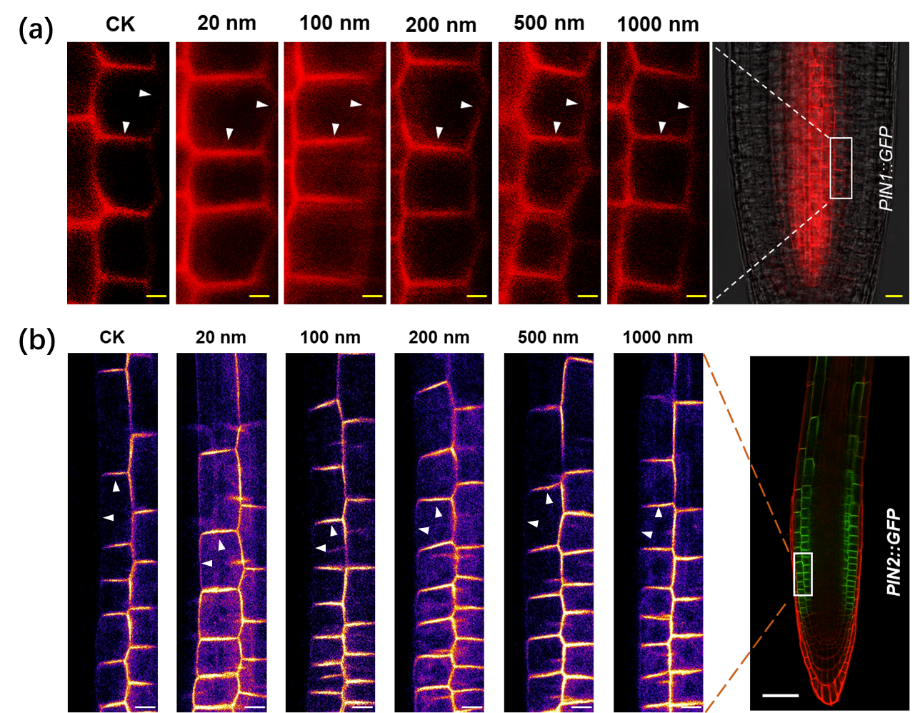


**Figure S20.** **Polar distribution of PIN2 proteins at the plasma membrane in root epidermal cells under different PS-NPs treatments.**

Cellular polarity analysis of auxin transporter proteins localized on plasma membranes in 4-day-old *pPIN2::PIN2-GFP* transgenic seedlings grown for 24 h in exposure to 40 μg/ml PS-NPs with various particle sizes. The white arrowheads indicate the two cellular sides for analysis. Scale bar 10 μm.


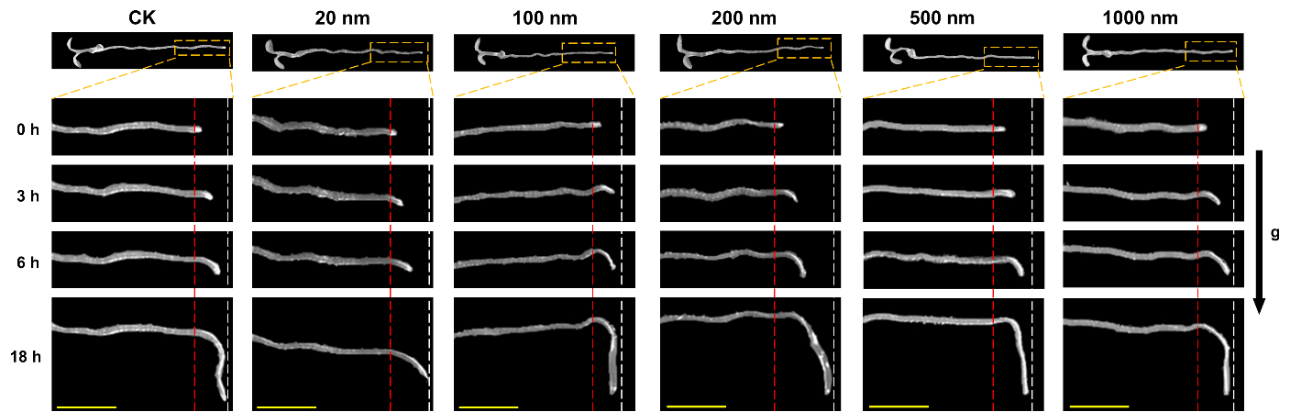


**Figure S21. Phenotypic analysis of plant root gravitropism under different PS-NPs treatments.**

The root gravitropic response of 5-day-old wild-type seedlings pre-grown in ^1^/_2_ MS medium containing 40 μg/ml of a series of particle size PS-NPs for 24 h at different time points. Black arrow indicates the direction of gravity. Scale bar 0.2 cm.


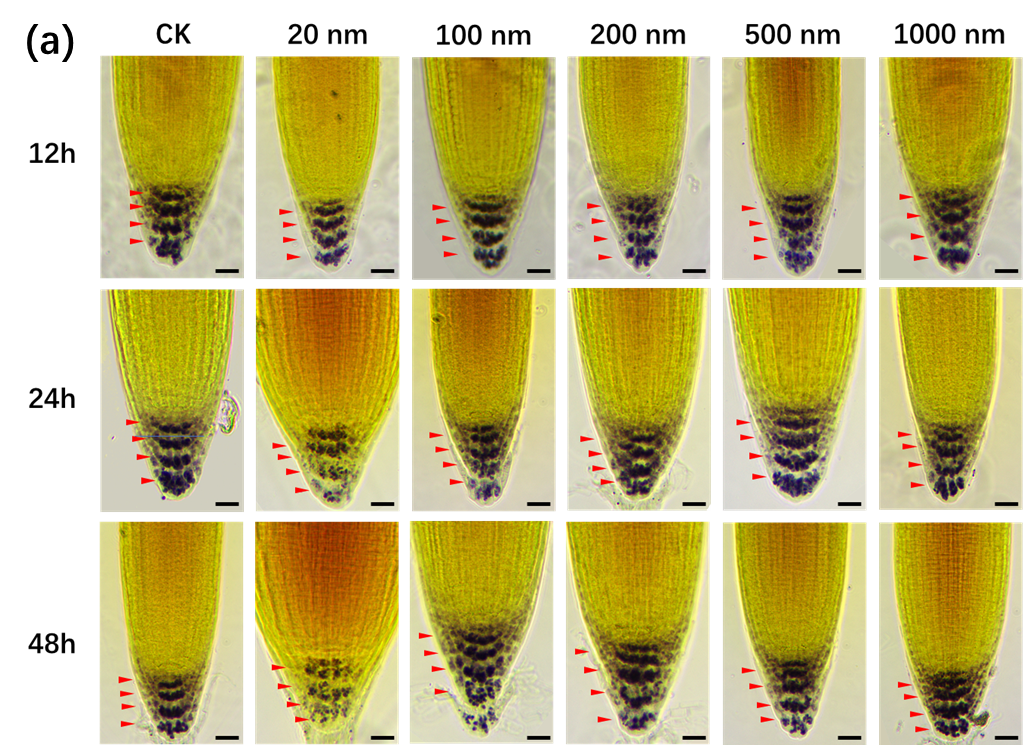


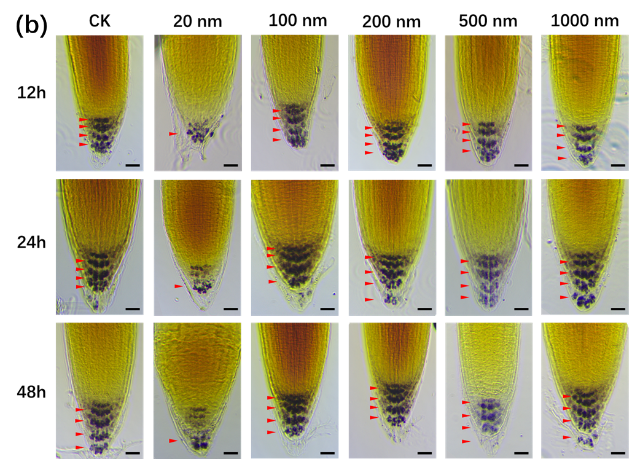


**Figure S22. Lugol’s staining illustrates starch granule accumulation within the root apex under different PS-NPs treatments.**

Accumulation of starch granules in root tips was observed under lugol’s staining in 4-day old wild-type seedlings grown for 48 h in ^1^/_2_ MS medium containing 40 μg/ml (a) or 100 μg/ml (b) PS-NPs with different particle sizes. The red arrowheads indicate the columella cells consisted with four cell layers. Scale bar 50 μm.


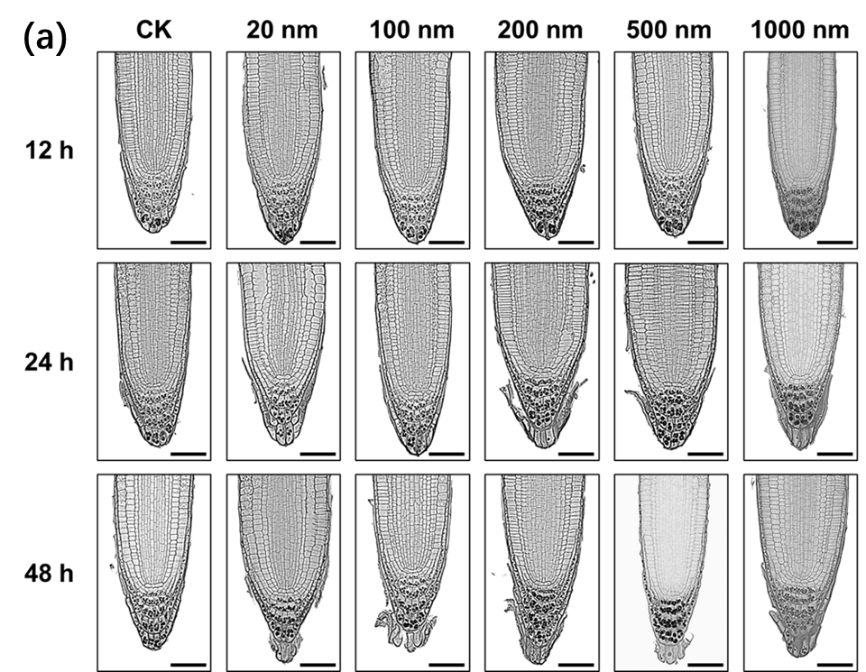

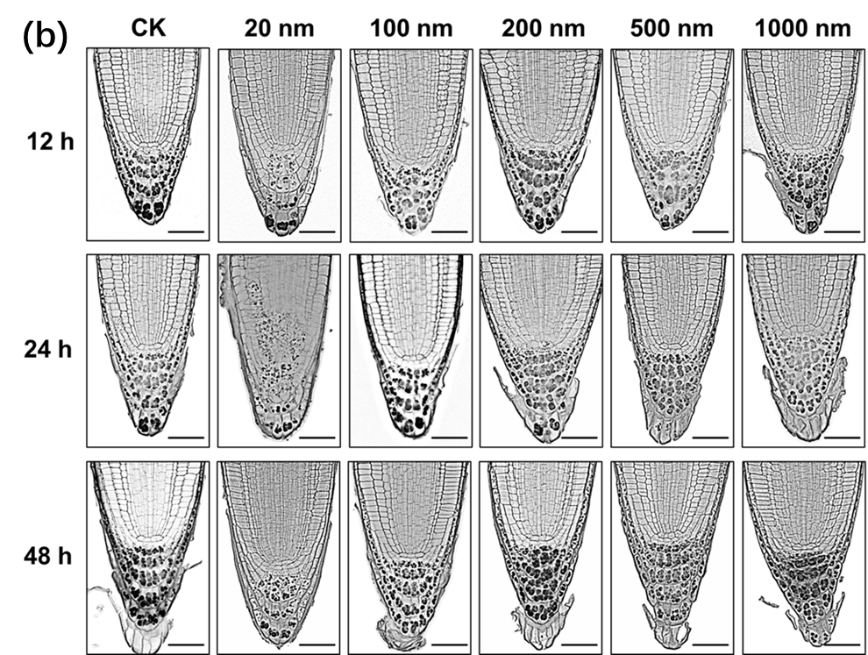


**Figure S23. The mPS-PI staining illustrates starch granule accumulation within the root apex under different PS-NPs treatments.**

The mPS-PI staining of starch granules in root tips of 4-day-old Col-0 seedlings grown in ^1^/_2_ MS medium containing 40 μg/ml (a) or 100 μg/ml (b) PS-NPs with different sizes for 48 h. Scale bar 50 μm.


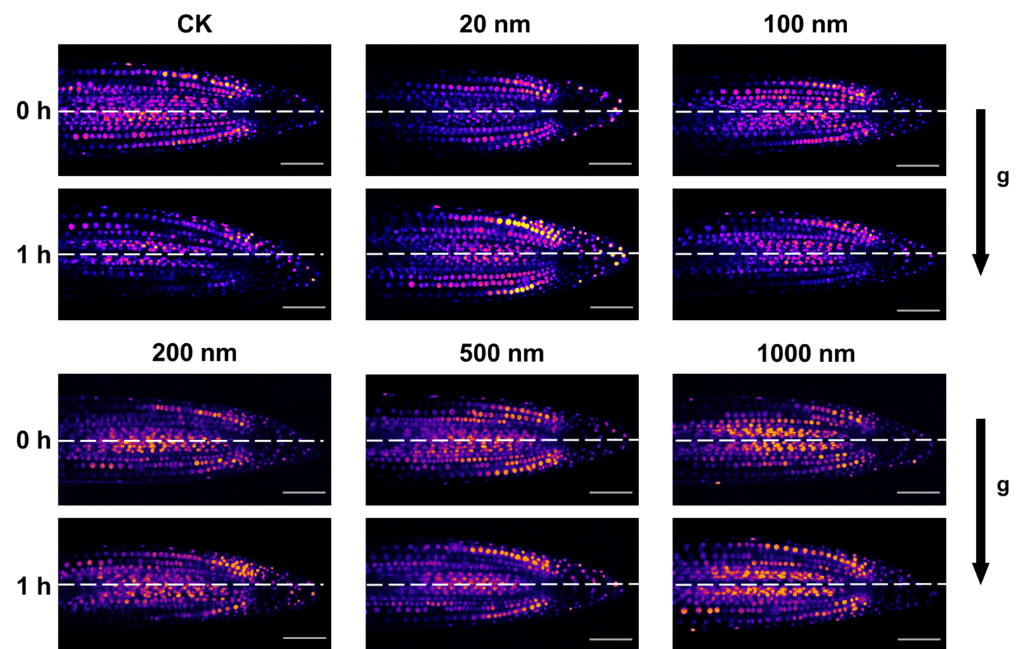


**Figure S24. Auxin redistribution was observed in *DⅡ::VENUS* treated with different PS-NPs, following 1 h of gravitropic stimulation.**

Representative CLSM images of the redistribution of Venus fluorescence signals on the upper and lower sides of the root tip after gravistimulation. 4-day-old *DⅡ::VENUS* transgenic seedlings grown on normal plates were transferred to plates containing 40 μg/ml PS-NPs with different sizes for continuing growth for 24 h and then rotated by 90 ° for 1 h. Black arrows indicate the direction of gravity. Scale bar 50 μm.


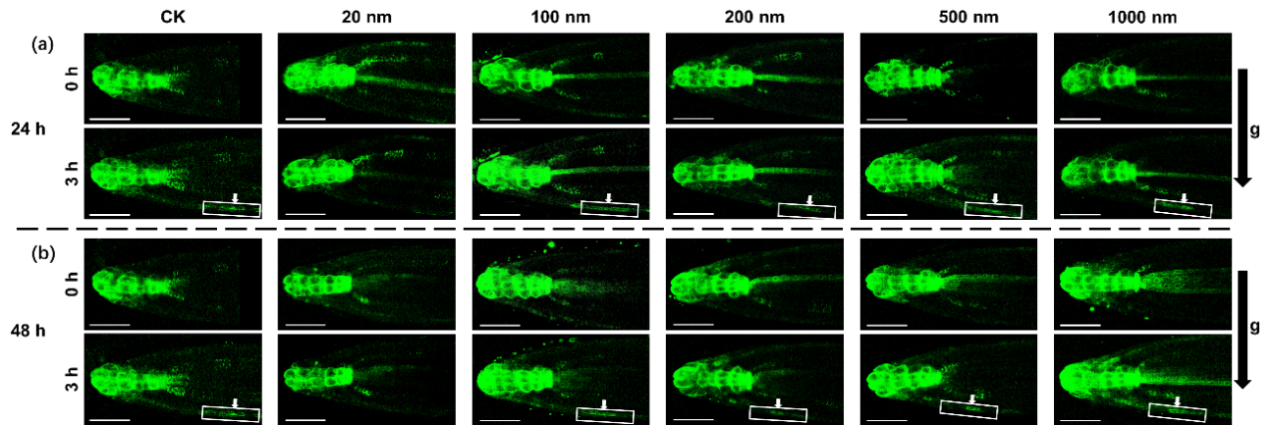


**Figure S25. Auxin redistribution in roots of synthetic auxin-responsive reporter *DR5rev::GFP* under different PS-NPs treatment after 3 h of gravistimulation.**

Small particle size PS-NPs inhibit the redistribution pattern of auxin under gravitational stimulation. 4-day-old *DR5rev::GFP* transgenic seedlings were transferred to ^1^/_2_ MS medium plates containing 40 μg/ml PS-NPs and continued to grow for 24 h (a) or 48 h (b), respectively, and then rotated by 90 ° for an additional 3 h. Root tips of GFP channels were imaged using laser confocal microscopy. Black arrows indicate the direction of gravity. Scale bar 100 μm.

**References**

1. Chen, S.; Zhou, Y.; Chen, Y.; Gu, J., fastp: an ultra-fast all-in-one FASTQ preprocessor. *Bioinformatics* **2018,** *34*, i884-i890.

2. Kim, D.; Langmead, B.; Salzberg, S. L., HISAT: a fast spliced aligner with low memory requirements. *Nat. Methods* **2015,** *12*, (4), 357-60

3. Anders, S.; Pyl, P. T.; Huber, W., HTSeq--a Python framework to work with high-throughput sequencing data. *Bioinformatics* **2015,** *31*, 166-9.

4. Roberts, A.; Trapnell, C.; Donaghey, J.; Rinn, J. L.; Pachter, L., Improving RNA-Seq expression estimates by correcting for fragment bias. *Genome Biol* **2011,** *12*, R22.

5. Love, M. I.; Huber, W.; Anders, S., Moderated estimation of fold change and dispersion for RNA-seq data with DESeq2. *Genome Biol* **2014,** *15*, 550.

6. Kanehisa, M.; Araki, M.; Goto, S.; Hattori, M.; Hirakawa, M.; Itoh, M.; Katayama, T.; Kawashima, S.; Okuda, S.; Tokimatsu, T.; Yamanishi, Y., KEGG for linking genomes to life and the environment. *Nucleic Acids Res.* **2008,** *36*, D480-484.
